# Simple Methods for Generating and Detecting Locus-Specific Mutations Induced with TALENs in the Zebrafish Genome

**DOI:** 10.1371/journal.pgen.1002861

**Published:** 2012-08-16

**Authors:** Timothy J. Dahlem, Kazuyuki Hoshijima, Michael J. Jurynec, Derrick Gunther, Colby G. Starker, Alexandra S. Locke, Allison M. Weis, Daniel F. Voytas, David Jonah Grunwald

**Affiliations:** 1 Department of Human Genetics, University of Utah, Salt Lake City, Utah, United States of America; 2 Department of Genetics, Cell Biology, and Development and Center for Genome Engineering, University of Minnesota, Minneapolis, Minnesota, United States of America; University of Pennsylvania School of Medicine, United States of America

## Abstract

The zebrafish is a powerful experimental system for uncovering gene function in vertebrate organisms. Nevertheless, studies in the zebrafish have been limited by the approaches available for eliminating gene function. Here we present simple and efficient methods for inducing, detecting, and recovering mutations at virtually any locus in the zebrafish. Briefly, double-strand DNA breaks are induced at a locus of interest by synthetic nucleases, called TALENs. Subsequent host repair of the DNA lesions leads to the generation of insertion and deletion mutations at the targeted locus. To detect the induced DNA sequence alterations at targeted loci, genomes are examined using High Resolution Melt Analysis, an efficient and sensitive method for detecting the presence of newly arising sequence polymorphisms. As the DNA binding specificity of a TALEN is determined by a custom designed array of DNA recognition modules, each of which interacts with a single target nucleotide, TALENs with very high target sequence specificities can be easily generated. Using freely accessible reagents and Web-based software, and a very simple cloning strategy, a TALEN that uniquely recognizes a specific pre-determined locus in the zebrafish genome can be generated within days. Here we develop and test the activity of four TALENs directed at different target genes. Using the experimental approach described here, every embryo injected with RNA encoding a TALEN will acquire targeted mutations. Multiple independently arising mutations are produced in each growing embryo, and up to 50% of the host genomes may acquire a targeted mutation. Upon reaching adulthood, approximately 90% of these animals transmit targeted mutations to their progeny. Results presented here indicate the TALENs are highly sequence-specific and produce minimal off-target effects. In all, it takes about two weeks to create a target-specific TALEN and generate growing embryos that harbor an array of germ line mutations at a pre-specified locus.

## Author Summary

Many genes are being discovered solely on the basis of their association with a trait or disease, or their relatedness to other known genes, but nevertheless the precise biological functions of these genes remain mysterious. We need new tools to discover the immediate molecular, cellular, and developmental functions of genes of interest. Increasingly, the zebrafish is being used as a model organism to discover gene functions that are shared among all vertebrates. In this study we develop new, highly efficient, and very easy to apply methods for generating zebrafish that lack the function of any desired gene. We also introduce sensitive and easy-to-apply methods for detecting newly arising mutations. The approach developed here can also be used to quickly eliminate the function of any chosen gene in other animals or in tissue culture cells. In all, we anticipate the methods described here will be widely applied to study gene function in many different contexts.

## Introduction

The zebrafish has emerged as a leading model organism for the study of vertebrate biology, because of the remarkable cellular resolution with which the embryo can be studied, the ease of assaying its development and physiology in the laboratory, and its amenability to genetic analyses. Forward genetic screens have been used to discover genes that contribute to tissue specification and morphogenesis, cell biology processes including growth regulation and genome maintenance, specificity of neural wiring, metabolism, behavior, and other aspects of the life cycle [1], [2], [3], [4], [5], [6], [7], [8]. Reverse genetics approaches have been used to uncover the biological processes controlled by genes of interest, and thus the zebrafish is being used increasingly to discover the immediate cellular and molecular functions of genes identified by virtue of their association with disease processes [9], [10], [11]. The methods developed here are aimed at improving and simplifying reverse genetics approaches in the zebrafish.

Several methods exist for perturbing the function of selected genes in the zebrafish, but until recently none reliably and efficiently eliminated the function of any specified gene [5], [12]. Gene function can be attenuated in the embryo with antisense morpholino oligonucleotides (MOs) [13], [14], but the rules for designing effective antisense oligonucleotides have not been perfected, the MOs themselves frequently have unintended off-target effects on development, and even when effective, MOs can only disrupt gene expression transiently [5], [15]. Therefore methods to isolate bona fide mutations in pre-selected genes continue to be pursued. Genome-screening methods have been used to identify and recover locus-specific mutations following random mutagenesis [16], [17], but although these methods are effective, they are highly labor-intensive and not very efficient.

In recent years the development of zinc finger nuclease (ZFN) technology portended the ability to induce mutations in any locus of any genome [18], [19], [20]. In this approach a double strand break (DSB) is induced at a unique site in the genome with a synthetic nuclease and host machinery repairs the chromosome break via the error-prone Non-Homologous End-Joining (NHEJ) pathway. Repair of such lesions often produces small insertions and/or deletions (indels) centered at the site of the DSB. Recent studies demonstrated that locus-specific mutations could be readily induced in zebrafish using ZFNs, and it appeared this approach might be applied to any locus [21], [22], [23]. However severe limitations still constrain the ability to generate zinc finger domains that can bind specifically to any desired genomic target sequence. Thus although ZFN-mediated targeted mutagenesis appeared promising, widespread implementation of the strategy requires a new approach for generating nucleases that exhibit very high sequence specificity.

The recent discovery of the Transcription Activator-Like Effector (TALE) proteins produced by plant pathogenic bacteria of the genus *Xanthomonas* has uncovered a new type of DNA-binding motif that can be used to create peptides that bind DNA with high affinity and sequence selectivity [24], [25], [26], [27]. TALE DNA binding is mediated by arrays of 33–35 amino acid DNA recognition motifs, each of which interacts with a single target nucleotide. As illuminated by X-ray crystallographic analysis of a TALE-DNA complex [28], [29], the process of DNA recognition occurs in a remarkably modular fashion, so that adjacent recognition motifs interact with adjacent nucleotides in a manner that does not appear to be affected by the presence of neighboring motifs. Nucleotide discrimination is determined by a pair of adjacent amino acid residues within the motif, called the Repeat Variable Di-Residue (RVD); hence the recognition motif is referred to as the RVD repeat module. Combining the simple modular TALE recognition cipher with a few empirically based guidelines, web-based algorithms have been established for designing RVD repeat-based DNA binding peptides that can bind genomic target sequences of interest [30].

Fusion of TALE-based DNA binding domains with the sequence-non-specific nuclease domain derived from the type IIS FokI restriction enzyme has been used to create sequence-specific nucleases, called TALENs [27], [31]. Preliminary studies have demonstrated the promise of TALENs for inducing locus-specific mutations in the zebrafish [12], [32]. Here we describe very simple methods, using reagents that are available for research without restriction, for generating TALENs that are extremely effective for inducing mutations at any locus in the zebrafish. The TALE-based DNA binding domains are generated quickly using a strategy that extracts sequences encoding RVD repeat modules from a library of plasmids and joins them in an ordered sequence using the Golden Gate cloning system [30]. We also establish easy and rapid methods for detecting the mutations induced by TALENs. Using the methods presented here, every embryo injected with mRNA encoding a TALEN will acquire mutations at the targeted locus in somatic tissues and approximately 90% of the animals that reach adulthood transmit newly induced specific locus mutations through their germ lines. In all, it takes about two weeks to create target-specific TALENs and generate growing embryos that harbor an array of germ line mutations at a pre-specified locus.

## Results

### Design and construction of TALENs

The TALENs we generate function as sequence-specific heterodimer endonucleases. Each monomer component is a chimeric protein composed of the FokI nuclease domain fused with a synthetic DNA binding domain consisting of an array of RVD repeat modules. Nuclease activity requires the binding of the two components on opposing strands of the duplex at a small interval distance. Although the FokI enzyme normally functions as a homodimer, we utilize mutant derivatives of the nuclease domain [23] so that the TALENs function as obligate heterodimers, thus demanding that both ‘Left’ and ‘Right’ monomers simultaneously recognize their cognate binding sites to achieve nuclease activity. The RVD repeat assembly reagents generated by Cermak et al. [30] allow construction of sequences encoding DNA binding domains composed of up to 31 recognition motifs. However, generally we design monomer TALEN components that each contain 16–20 RVD repeats and that, including the DNA interaction function of the N-terminal portion of the TALEN [28], [29], bind a half-target site of approximately 17–21 nt present on opposing strands and spaced apart by 14–17 bp (target site configuration ≥17 bp – N_14–17_ – ≥17 bp). Applying parameters described in Materials and Methods to the TALEN Targeter program (https://boglab.plp.iastate.edu/node/add/talen), TALENs can be designed that recognize only a single target site in the zebrafish genome. Such unique target sites can be identified in many exons, as well as introns and promoter sequences (see Discussion). The gene sequences targeted and the RVD repeat arrays of the TALENs used in this study are presented in Figure S1.

To generate a plasmid encoding a Left or Right monomer component consisting of *n* RVD repeats (see Materials and Methods for details), an initial Golden Gate cloning step is used to assemble two arrays, encoding repeats 1–10 and repeats 11 – *n-1*, as in [30]. The final expression plasmid encoding an entire TALEN monomer is generated in a second Golden Gate cloning assembly, which brings together the two partial arrays, sequences encoding the *n^th^* motif, and a modified CS2^+^ backbone vector, pCS2TAL3RR or pCS2TAL3DD (Figure S2; see Materials and Methods). Assembly results in a fusion gene that encodes: vector-provided N-terminal TALE-derived sequences, an RVD repeat array, C-terminal TALE-derived sequences, and a modified nuclease domain. TALENs can be expressed directly from the CMV promoter resident in these CS2^+^ vectors. However, for the zebrafish experiments described below, mRNA encoding Left or Right monomer components were generated individually by *in vitro* transcription of linearized plasmids and equal amounts of each mRNA were co-injected into embryos at the 1 cell stage.

### Induction of somatic mutations at the *golden* locus

**Figure 1:**
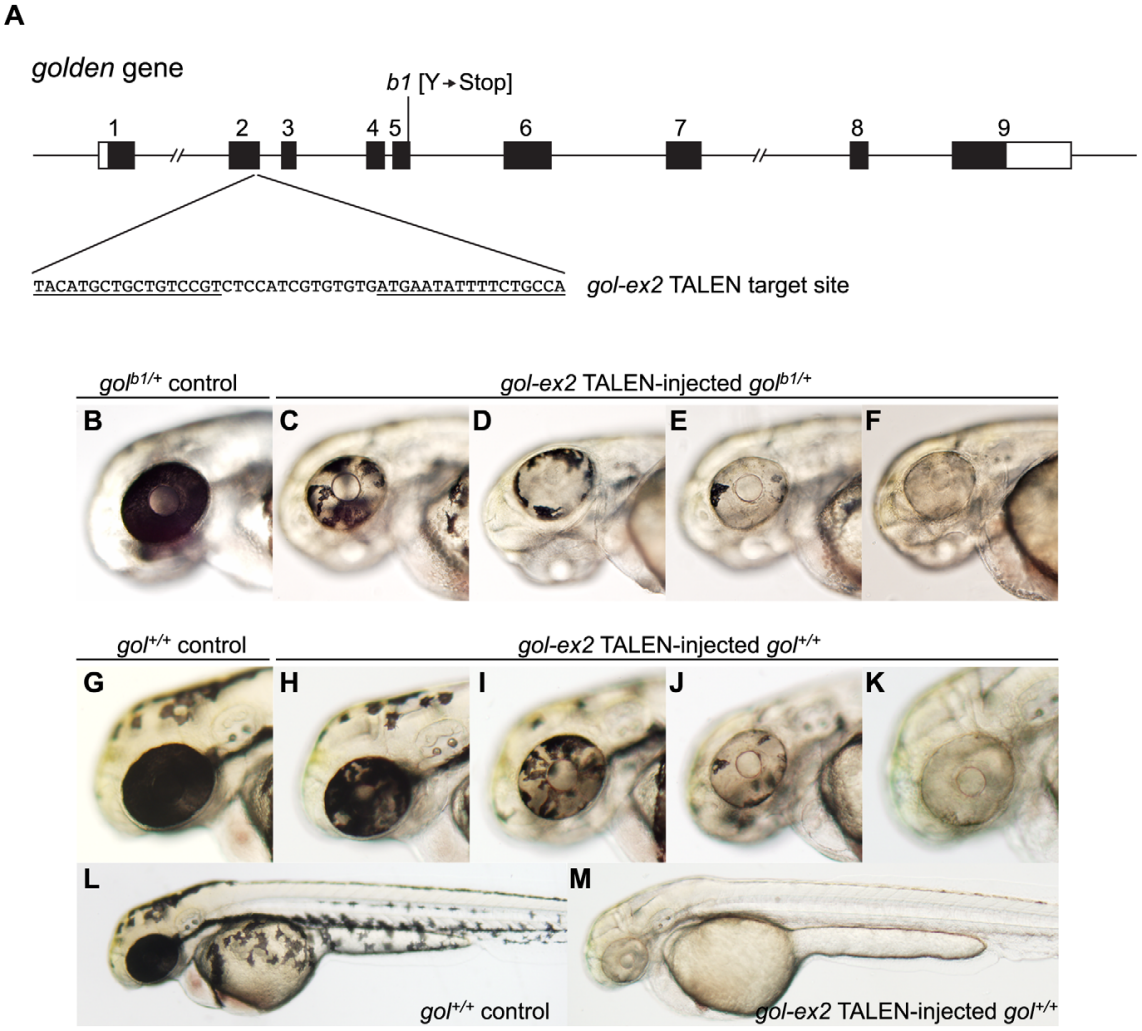
Induction of somatic *golden* mutations with TALENs. (A) Schematic representation of the genomic structure of the *golden* (*gol*) gene, with coding and untranslated exon regions depicted as solid and open boxes, respectively. The locations of the *gol-ex2* TALEN target site in exon 2 and the *b1* null mutation in exon 5 [33] are indicated. The *gol-ex2* TALEN target sequence, with Left and Right TALEN monomer binding sites underlined, is shown. (B–F) Induction of *golden* mutant cells in the Retinal Pigmented Epithelium (RPE) of heterozygous *gol^b1/+^* embryos. Whereas the entire RPE of a 2 dpf heterozygous *gol^b1/+^* control embryo (B) is darkly pigmented, the RPEs of *gol^b1/+^* embryos injected with *gol-ex2* TALEN RNAs (100 pg total) had patches of pigmentless mutant tissue (C–F). (G–M) Induction of *golden* mutant cells in the soma of wildtype *gol^+/+^* embryos. Wildtype *gol^+/+^* control embryos have darkly pigmented RPEs (G) and dark melanophores scattered over their bodies (L). Following injection at the 1 cell stage with 100 pg *gol-ex2* TALEN RNAs, *gol^+/+^* embryos had patches of pigmentless *golden* mutant tissue in the RPEs (H–K) and some injected embryos appeared entirely devoid of pigmentation (M).

To estimate the efficiency with which targeted mutations can be induced, we measured the ability of TALENs to induce somatic tissue mutations in the *golden (gol)* gene, which governs pigmentation in the embryo and adult without compromising viability [33], [34]. Mutations at *gol* are recessive: embryos with at least one WT allele are darkly pigmented at 2 days postfertilization (dpf), whereas homozygous *gol* mutants appear pigmentless at this stage. To maximize the chance of inducing complete loss-of-function alleles, we chose to develop a TALEN that would generate DSBs within coding sequences residing toward the 5′ end of the *gol* locus. Using criteria described in Materials and Methods, a potential TALEN target site was identified in the second exon of *gol* and the *gol-ex2* TALEN was designed (Figure 1A, Figure S1). As imprecise repair of targeted DSBs is likely to produce recessive loss-of-function mutations at *gol*, we injected embryos heterozygous for the *gol^b1^* null mutation [33], [34] with *gol-ex2* TALEN mRNA and measured the appearance of gol^b1/*^ mutant pigmentless cells in the Retinal Pigmented Epithelium (RPE) (Figure 1). At 2 dpf the RPE is a monolayer of approximately 550 pigmented cells that envelops each eye, and the presence of even small clones of pigmentless tissue can be detected easily [35]. Whereas it is extremely rare for pigmentless cells to be found in the RPEs of control *gol^b1/+^* heterozygous embryos [36], all but one of 20 TALEN-injected *gol^b1/+^* embryos had large patches of *gol* mutant cells (Figure 1B–1F, Table 1). The TALEN-injected embryos had multiple patches of mutant tissue, indicating they were genetically mosaic.

**Table 1:** Induction of *golden* mutant cells by the *gol-ex2* TALEN.

Host embryo Genotype	Amount TALEN RNA injected	# embryos	# (%) embryos with *golden* cells
*gol^b1/+^*	Uninjected	37	0
	100 pg	20	19 (95.0%)
*gol^+/+^*	Uninjected	295	0
	4 pg	92	38 (41.3%)
	20 pg	79	71 (89.9%)
	100 pg	275	271 (98.5%)

One cell stage embryos were injected with *gol-ex2* TALEN mRNAs as indicated and the appearance of clones of pigmentless cells was scored in the 2 dpf RPE.

On average ≥50% of the RPE cells in TALEN RNA-injected embryos were *golden* (Figure 1C–1F), indicating the majority of genomes in the embryos acquired TALEN-induced mutations. Three experiments demonstrated the new mutations were indeed induced by the *gol-ex2* TALEN. First, the *gol-ex2* TALEN could induce pigmentless tissue in the RPEs of *gol^+/+^* embryos (Figure 1G–1K), indicating the induction of mutant tissue did not require a pre-existing *gol* mutant allele. Mutant cells were observed in almost 100% of these injected WT embryos (Table 1) and occasionally even wholly *gol* embryos were observed (Figure 1L, 1M; see Table S2 for frequencies), highlighting the efficiency with which these TALENs can induce mutations in both genomes of a cell. Second, as discussed below, analysis of exon 2 sequences amplified from *gol-ex2* TALEN RNA-injected embryos revealed a diverse set of indel mutations were induced, typical of those produced by NHEJ-mediated repair of DSBs. Third, *gol* mutant alleles were transmitted to the F1 offspring of TALEN-injected WT embryos (see below). We conclude the *gol-ex2* TALEN is extremely effective at inducing mutations at *gol*.

### A general strategy for detecting mutations induced with TALENs

Whereas newly induced mutations at *golden* are simple to detect, TALEN-induced mutations at most loci are unlikely to present a phenotype that can be scored in individual somatic cells. As the repair of DSBs can lead to an assortment of indels centered at the TALEN target site, it is desirable to detect induced mutations with a method that can detect any DNA change arising at a selected target site. Furthermore, as DSBs are induced only following translation of injected TALEN mRNAs, an individual embryo may acquire several independent mutations, each of which may arise in a mosaic fashion during development. Therefore a sensitive method is required that can detect mixtures of TALEN-induced DNA polymorphisms at a targeted locus present among the genomes of a single embryo.

**Figure 2:**
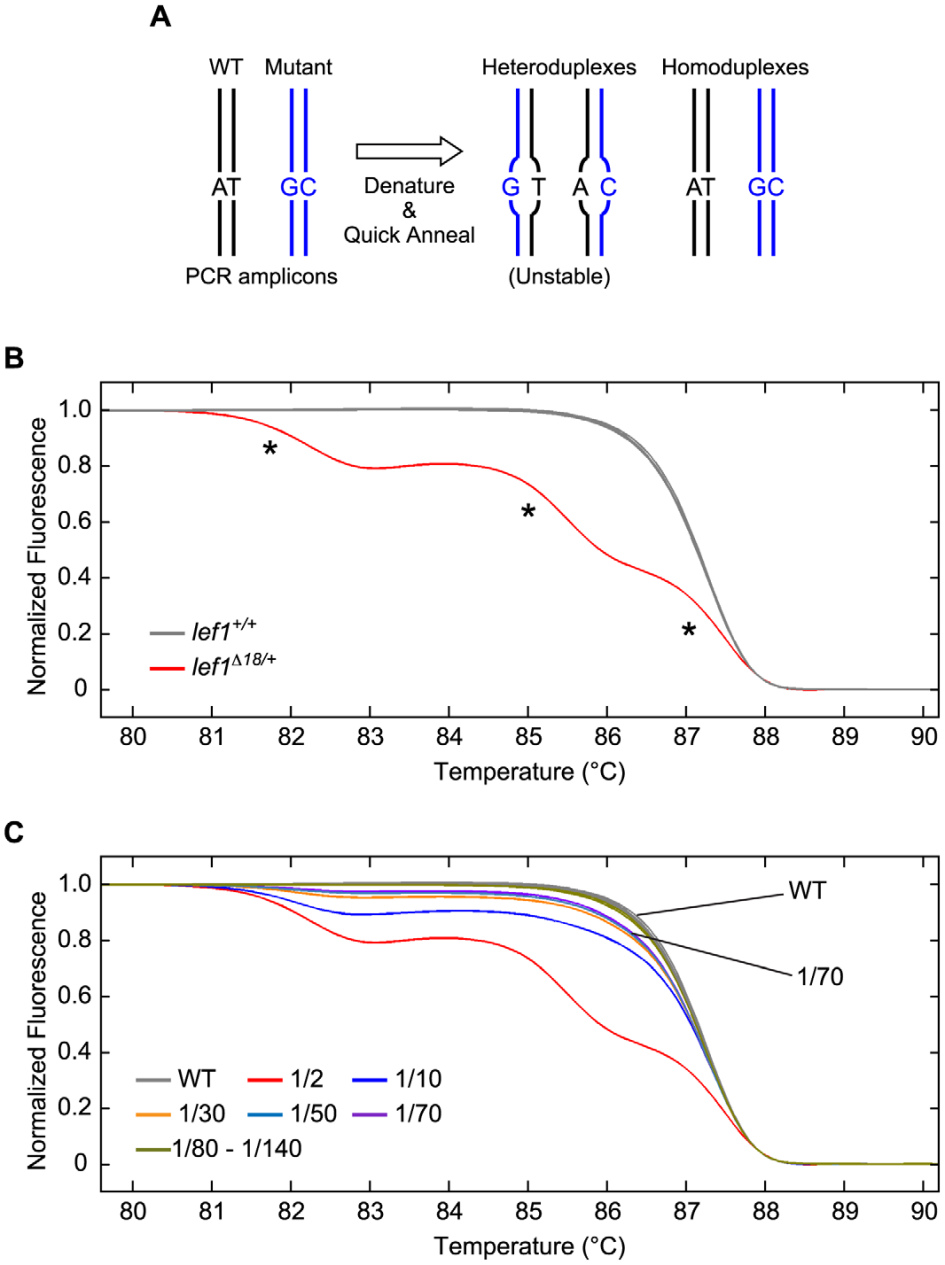
HRMA detects the presence of mutant alleles in mixed populations of wildtype and mutant genomes. (A) Schematic illustration of the principle of HRMA. A small region of the genome that includes a DNA polymorphism is amplified by PCR from a mixed population of wildtype (WT) and mutant template genomes. Heat denaturation of the amplicons followed by rapid re-annealing of DNA strands produces two kinds of homoduplex and two kinds of heteroduplex products. Heteroduplex products are likely to be particularly unstable and exhibit a Tm that differs from the homoduplex products. (B) HRMA of *lef1* PCR amplicons generated from template genomes that were either WT (*lef1^+/+^*) or heterozygous for an 18 bp deletion (*lef1^Δ18/+^*). PCR amplifications were performed in the presence of LC Green Plus dye (Idaho Technology), which fluoresces upon binding double strand DNA, allowing the detection of intact duplex molecules. The DNAs amplified from the *lef1^+/+^* genome (grey curves) comprise a homogeneous population of duplexes with a single Tm. In contrast, re-annealed amplicons derived from the *lef1^Δ18/+^* genome (red curve) are composed of multiple duplex populations, which display distinct Tms (*). (C) Sensitivity of HRMA for detecting the presence of the *lef1^Δ18^* mutation in mixtures of *lef1^+/+^* WT and *lef1^Δ18/+^* heterozygous genomes. Heterozygous DNA was mixed with increasing amounts of WT DNA; fractions indicate the relative abundance of mutant haploid genomes in the mixtures. A constant total amount of genomic template DNA and primers was used to generate each set of amplicons. LightScanner Call-IT Software (Idaho Technology) was used to identify melt curves that differed significantly from WT. HRMA detected the *lef1* 18 bp deletion allele when it was present as only 1/70^th^ of the total haploid genomes.

We find High Resolution Melt Analysis (HRMA) is a simple, rapid, and sensitive method for detecting TALEN-induced somatic mutations. HRMA has been used in the past to detect known sequence polymorphisms in the zebrafish [37], but it is also useful for discovering polymorphisms of unknown sequence that lie in a small, defined region of the genome. We developed standard conditions for detecting sequence alterations resulting from TALEN activity at targeted loci (Materials and Methods). To detect the occurrence of a DNA polymorphism at a particular locus, short PCR amplicons (90–120 bp) that include the region of interest are generated from a genomic DNA (gDNA) sample, subjected to denaturation and rapid renaturation, and the thermostability of the population of renatured amplicons is analyzed (Materials and Methods). If TALEN-induced polymorphisms are present in the template gDNA, heteroduplex as well as different homoduplex molecules will be formed (Figure 2A). The presence of multiple forms of duplex molecules is detected by HRMA, which records the profile of temperature-dependent denaturation and detects whether duplex melting acts as a single species or more than one species. For example, three prominent duplex types with distinct melting temperatures (Tm's) are evident in the analysis of renatured *lef1* amplicons generated from an embryo heterozygous for an intragenic 18 bp deletion in *lef1* (Figure 2B).

**Figure 3:**
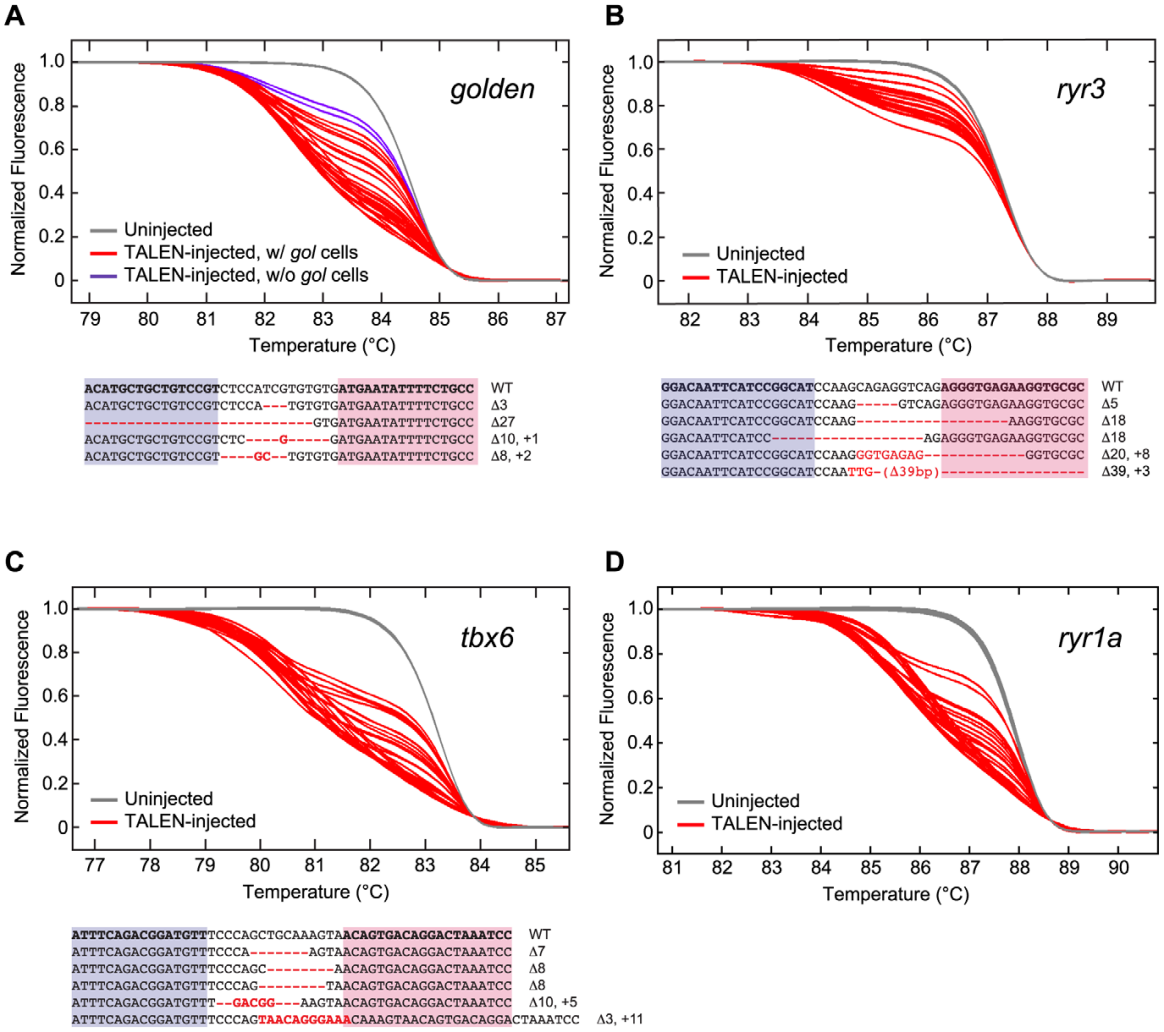
Induction of somatic mutations with TALENs. (A–D, Upper panels) HRMA detection of targeted mutations in WT embryos that had been injected at the 1 cell stage with RNAs encoding TALENs directed against the *golden* (A), *ryr3* (B), *tbx6* (C), or *ryr1a* (D) gene (see Figure S1 for target sites). gDNA was isolated from individual uninjected or TALEN RNA-injected 1–2 dpf embryos and subjected to HRMA. Each curve is the melting profile of re-annealed amplicons generated from a single embryo. LightScanner Call-IT Software (Idaho Technology) was used to identify melt curves that differed significantly from WT. The results shown here reflect a single experiment, but results from all injections are tablulated in Table 2. Newly induced DNA polymorphisms at the targeted loci were evident in all but one injected embryo (red curves, Table 2), and even *gol-ex2* TALEN RNA-injected embryos that did not exhibit patches of pigmentless tissue had induced *golden* mutations as detected by HRMA (A, purple curves). (A–C, Lower panels) TALEN-induced sequence alterations at targeted loci. Genomic sequences bordering the targeted loci were amplified from embryonic gDNA samples, cloned, and sequenced. Examples of recovered alleles (purple and pink boxed sequences indicate Left and Right RVD binding sites of the TALENs, respectively; red indicates sequence alterations) indicate that insertion/deletion (indel) mutations centered at the TALEN target sites had been induced in somatic tissues of embryos.

We determined the sensitivity of HRMA for detecting the presence of a mutant genome mixed among a population of WT genomes using standard conditions of analysis (Materials and Methods). gDNA prepared from an embryo heterozygous for the *lef1^Δ18^* mutation was mixed with differing amounts of WT gDNA, and the ability of HRMA to detect the mutant allele was determined. As the mutant genome is present as a decreasing fraction of all template genomes, the relative abundance among amplicons of homoduplex mutant and heteroduplex populations changes, but the presence of mutant genomes can be detected unambiguously even when mutant genomes represent 1/70^th^ of the total mix (Figure 2C). Figure S3 shows a 4 bp insertion mutation in the *lef1* gene can be detected with similar sensitivity. In genetically mosaic embryos, when multiple mutant alleles are present as minor populations within a mixture of genome, the melt curves become more complex, but deflections away from the WT profile are additive and therefore simple to detect (see examples in Figure 3). Given our findings (presented below) that the majority of mutations induced using the methods described here consist of alterations of >4 bp, we estimate our standard conditions of analysis can routinely detect one mutant genome present among 50 WT genomes.

### HRMA reveals TALENs induce mutations efficiently at many loci

To test the efficacy of our methods for generating and detecting TALEN-induced somatic mutations at any locus, we injected 1 cell embryos with mRNAs encoding the *gol-ex2* TALEN or TALENs designed to recognize and cleave sequences in exon 3 of *tbx6*, exon 5 of *ryr3*, or exon 6 of *ryr1a* (Figure S1). Under standard conditions of injection with 100 pg total TALEN RNA, approximately 95% of the embryos developed normally (Table S3). gDNA was prepared from individual 1–2 dpf TALEN-injected or control embryos and analyzed by HRMA for the presence of DNA sequence variants at the targeted loci using primer pairs listed in Table S1. Nearly all TALEN RNA-injected embryos had targeted mutations, including the *gol-ex2* TALEN RNA-injected embryos that did not have *gol* mutant cells in the RPE (Table 2, Figure 3). Sequence analysis of PCR amplicons covering the targeted loci (Figure 3) indicated the induction of a spectrum of indel mutations, consistent with what is expected from NHEJ repair. Analysis of loci amplified from 9 embryos targeted at *tbx6*, *ryr3*, or *ryr1a* indicated approximately half the genomes (26/52) of TALEN-injected embryos harbored targeted mutations.

**Table 2:** Induction of DNA sequence alterations at targeted loci.

Targeted locus	Amount TALEN RNA injected	# embryos	# (%) embryos with mutations
*golden* [exon 2]	Uninjected	7	0 (0%)
	100 pg	42	42 (100%)
*ryr3* [exon 5]	Uninjected	16	0 (0%)
	100 pg	47	46 (97.9%)
*tbx6* [exon 3]	Uninjected	25	0 (0%)
	100 pg	54	54 (100%)
*ryr1a* [exon 6]	Uninjected	24	0 (0%)
	100 pg	24	24 (100%)

One cell stage embryos were injected with TALEN mRNAs as indicated. gDNA from individual 1–2 dpf embryos was analyzed by HRMA for sequence alterations at targeted loci.

### Dose-dependent induction of mutations

**Figure 4:**
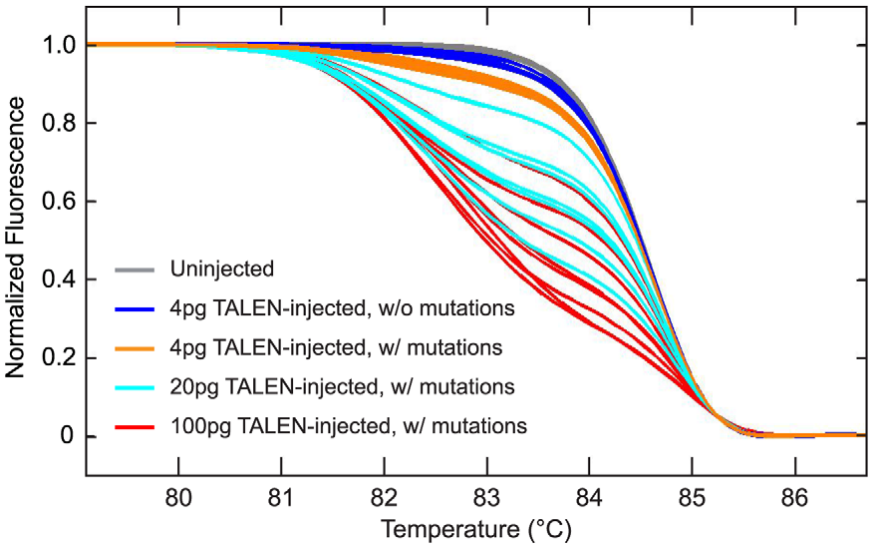
Dose-dependent induction of mutations with TALENs. WT embryos were either not injected or injected at the 1 cell stage with 4 pg, 20 pg, or 100 pg total *gol-ex2* TALEN RNA. gDNA was prepared from individual 1 dpf embryos and analyzed by HRMA for the presence of targeted mutations. Every embryo injected with 20 or 100 pg total RNA had mutations, but some 4 pg RNA-injected embryos did not have clear evidence of induced mutations (dark blue). As the amount of injected RNA was increased, the melt profiles of injected embryos, on average, diverged increasingly from the WT curves, indicating that increasing amounts of TALENs produce increasing numbers of mutant genomes.

As *gol* does not encode an essential function, the induction of *gol* mutant cells is not detrimental to development. However for other targeted genes it may be advantageous to avoid inducing bi-allelic mutations in many cells of the embryo. The frequency with which mutant cells are induced is dependent on the amount of TALEN RNA that is injected (Table 1, Table S2), so the frequency of cells harboring bi-allelic mutations can be adjusted. As shown in Figure 4, HRMA can be used to determine the dose-dependent induction of mutations that cannot be easily measured in somatic tissues. The amount of TALEN RNA delivered to embryos affects both the fraction of embryos that harbor detectable mutations as well as the average abundance of mutant genomes present in any individual injected embryo.

### Recovery of TALEN–induced germ line mutations

**Figure 5:**
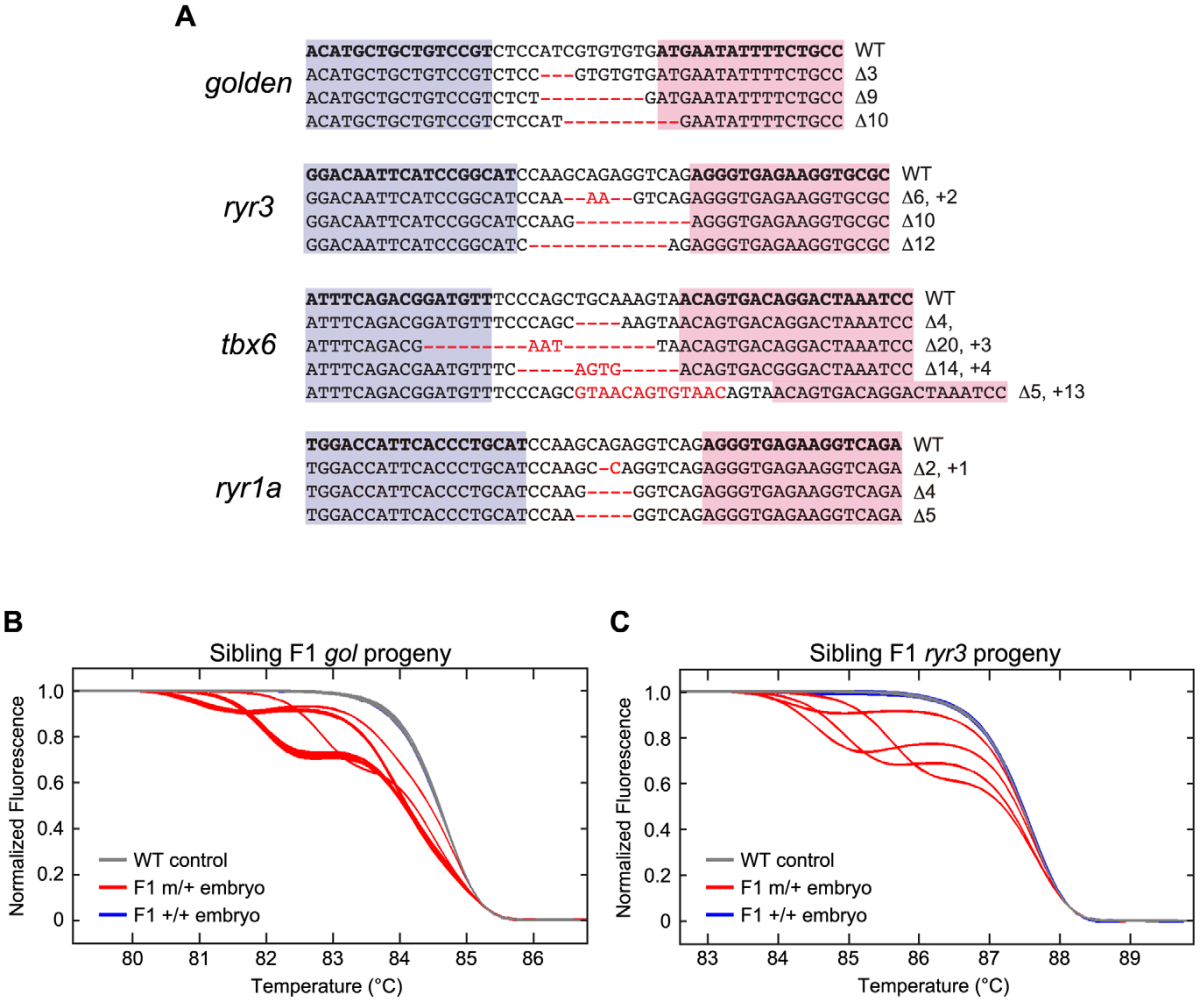
Induction of germ line mutations with TALENs. (A) TALEN RNA-injected G0 founders transmit targeted mutations to their offspring. G0 founders were mated with WT partners and the gDNAs of individual 1 dpf F1 progeny were examined by HRMA for the presence of mutations. The region flanking targeted sites was cloned and sequenced following PCR amplification from the gDNA of F1 offspring. Examples of germ line-transmitted mutations at each targeted locus are shown (purple and pink boxed sequences indicate Left and Right RVD binding sites of the TALENs, respectively; red indicates sequence alterations). (B, C) The germ lines of individual G0 founders are mosaic and can harbor several targeted mutations. Sibling F1 progeny of a single G0 founder targeted at *gol* (B) or a single G0 founder targeted at *ryr3* (C) were examined by HRMA for inheritance of new mutations at *gol* or *ryr3*, respectively. Some F1 embryos (*+/+*) had no mutations, as their HRMA profiles (blue) resembled that of control WT embryos (grey). Other F1 embryos (*m/+*) displayed HRMA profiles indicating heterozygosity for a newly induced mutant allele (red). Several distinct HRMA profiles were identified among the heterozygous F1 progeny of a single G0 founder, indicating multiple mutations were transmitted by each G0 founder.

To determine if induced mutations detected in 1–2 dpf embryos enter the germ line, we raised TALEN-injected (G0) embryos to adulthood and analyzed the transmission of mutations to progeny. Individual adult G0 animals were mated with WT partners and gDNA isolated from individual F1 embryos was analyzed by HRMA. Targeted germ line mutations were transmitted by 51 of the 57 G0 animals (approximately 90%) that had been exposed to TALENs directed at the *golden*, *tbx6*, *ryr3*, or *ryr1a* genes (complete transmission data is provided in Tables S4, S5, S6, S7; data is summarized in Table 3). The fraction of G0s carrying germ line mutations induced by each TALEN ranged from 77% to 100%. Analysis of the sequence alterations inherited by F1 embryos revealed the range of induced indel mutations among the germ line transmitted mutations mimicked those that had been observed in embryos (Figure 5A). The majority of TALEN-induced mutations identified here in injected G0, 1–2 dpf F1, or adult F1 individuals were sequence changes of 3–20 bp (Figure 3, Figure 5, Table S8). Larger indels were identified, but rarely. Most if not all mutations identified among the 1 dpf F1 offspring were viable in the heterozygous state, as adult F1s descended from G0 founders harbored a distribution of mutations similar to that found among the F1 embryos (Table S8). To date all the F1 fish heterozygous for TALEN-induced mutations at *tbx6*, *ryr1a*, and *ryr3* that have been bred (n = 16) have transmitted their mutations to the F2 generation. We have no evidence of mutations in the F1 generation that could not be further propagated.

**Table 3:** Distribution of germ line mutations induced by TALENs.

		Among G0 founders that transmit mutations	
Targeted locus	Fraction G0 founders with germ line mutations	Percent each G0 germ line with a targeted mutation*	Mean # of mutant alleles per G0 germ line
*golden* [exon 2]	14/15 (93.3%)	41.8% (4.2–100%)	2.29
*ryr3* [exon 5]	17/22 (77.3%)	18.0% (4.2–37.5%)	1.17
*tbx6* [exon 3]	11/11 (100%)	30.6% (16.7–50%)	2.00
*ryr1a* [exon 6]	8/9 (88.9%)	44.0% (4.2–100%)	2.50

To detect transmission of germ line mutations, TALEN RNA-injected G0 embryos were raised to adulthood and mated with WT partners to generate F1 offspring. gDNAs of individual 1–2 dpf F1 progeny were analyzed by HRMA for sequence alterations at targeted loci. Complete data for all G0 germ lines analyzed are presented in Tables S4, S5, S6, S7. G0 founders that transmitted mutations were further analyzed to determine the number of different mutations transmitted by each germ line and the fraction of gametes of each germ line that transmitted a mutant allele. The mean number of mutant alleles per G0 germ line was estimated based on the number of distinct HRMA melt patterns observed among the F1 progeny of each G0 founder. As analysis of the G0 germ lines was not exhaustive, the mean number of mutant alleles per G0 germ line is likely an underestimate.

* The Mean and (Range) are presented.

Most G0 animals transmitted multiple mutant alleles and most mutations were transmitted by significantly less than 50% of the gametes (Table 3; Tables S4, S5, S6, S7, S8), indicating the germ lines of G0 founders were mosaic. HRMA analysis of heterozygous F1 offspring was used to distinguish transmitted mutant alleles, and sibling individuals that appeared to carry a common DNA sequence alteration were grouped based on common melt curve shapes. As illustrated in Figure 5B and 5C, individual sibling F1 embryos descended from a single mutagenized G0 founder could harbor different alleles, indicated by the multiple distinct HRMA melting profiles of amplicons derived from sibling F1 offspring. On average, two new alleles were recovered from the germ lines of each mutagenized G0 founder (Table 3).

The finding that the germ lines of most G0 founders were genetically mosaic is consistent with the interpretation that TALENs induce mutations independently in different cells of the embryo and mutations accrue as the embryo develops. Indeed we found that following injection of 1 cell stage embryos with *gol-ex2* TALEN mRNAs, the fraction of embryos with detectable levels of targeted mutations increased with developmental time (Figure S4). Whereas 100% of injected embryos had mutations at 6 or 24 hours postfertilization (hpf), only a fraction of the 3 hpf embryos had detectable mutations, and among the early stage embryos, HRMA genotype analyses indicated a relatively low abundance of mutant genomes (Figure S4). Altogether these data indicate numerous targeted indel mutations can be recovered routinely from a small set of adults arising from TALEN mRNA-injected embryos.

### TALENs demonstrate target locus specificity

The very high frequency with which mutations were induced raised the possibility that the TALENs were simply mutagenic in a sequence-non-specific manner. However, in contrast with published reports [21], [22], [23] and our own experience with ZFNs (data not shown), TALENs exhibit very low toxicity. For example, although injection of only 4 pg total RNA encoding any of the TALENs used here was sufficient to induce mutations in the majority of embryos (Figure 4 and data not shown), approximately 95% of the embryos developed normally even after being injected with 100 pg total TALEN RNA (Table S3). These observations are consistent with the interpretation that random chromosome breaks are generally not produced by the TALENs.

**Figure 6:**
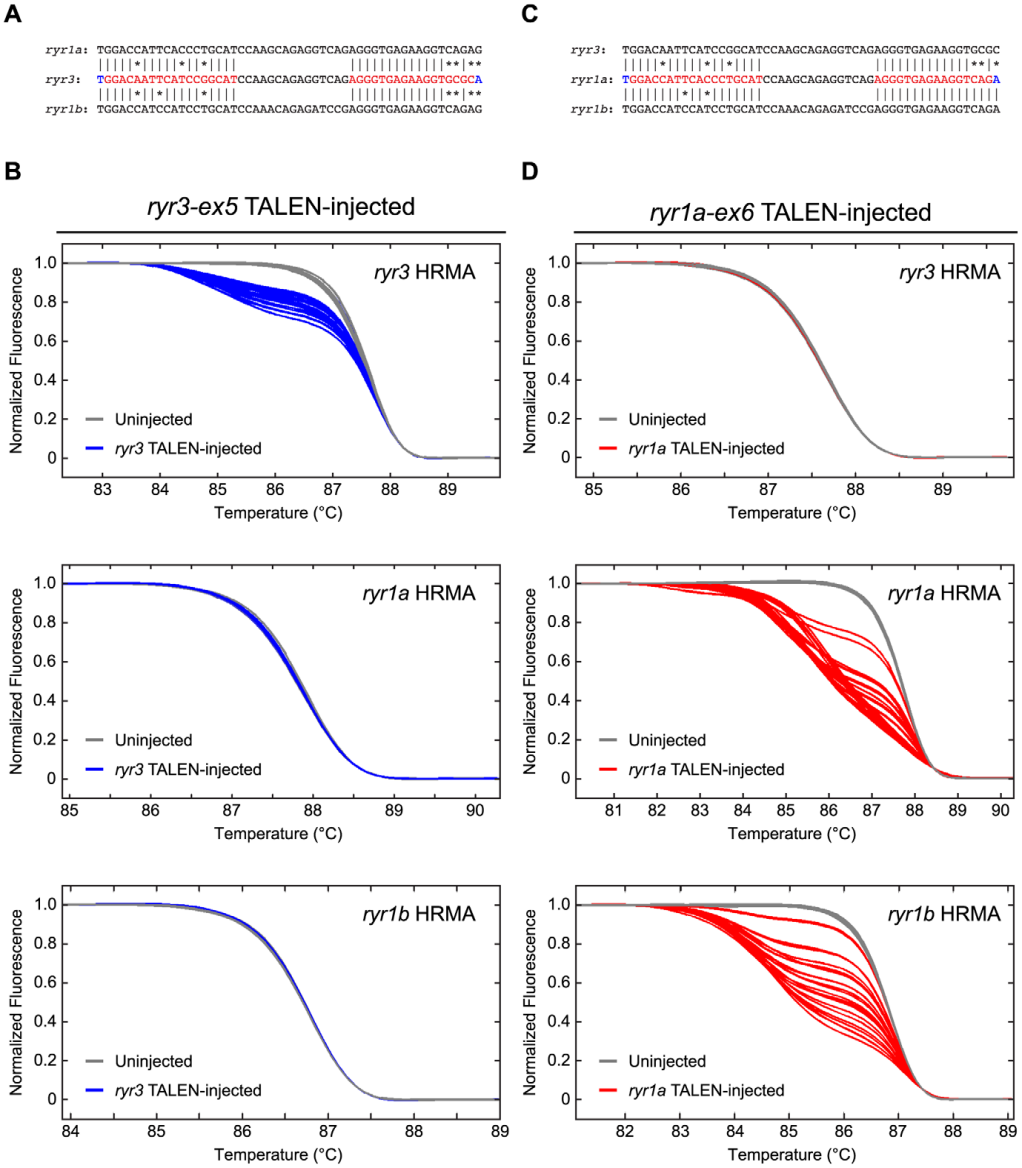
TALEN activity at homologous target sequences. (A) Comparison of the *ryr3* gene sequences targeted by the *ryr3-ex5* TALEN and homologous sequences of the *ryr1a* and *ryr1b* genes. *ryr3* sequences bound by the *ryr3-ex5* TALEN are denoted in color, as in Figure S1: Red letters indicate the Left and Right RVD array binding sites of the TALEN monomer components; the entire target site is defined as including both the nucleotides recognized by RVD array and the 5′ T that interacts with the N-terminal portion of the TALEN. Vertical lines indicate bases that are identical between the *ryr3-ex5* TALEN target and the gene sequences of homologues; *'s indicate non-identical bases. (B) Uninjected WT embryos (n = 23, grey curves) and WT embryos injected with 100 pg total *ryr3-ex5* TALEN RNAs (n = 24, blue curves) were analyzed by HRMA at 1 dpf for the presence of newly induced mutations. The gDNA of each embryo was examined in parallel for mutations at *ryr3*, *ryr1a*, or *ryr1b*. Whereas every injected embryo had *ryr3* mutations, no mutations were detected in the *ryr1a* or *ryr1b* genes. (C) Comparison of the *ryr1a* gene sequences targeted by the *ryr1a-ex6* TALEN and homologous sequences of the *ryr3* and *ryr1b* genes. *ryr1a* sequences bound by the *ryr1a-ex6* TALEN are denoted in color, as in (A). Identical and non-identical bases are indicated as in (A). (D) Uninjected WT embryos (n = 23, grey curves) or WT embryos injected with 100 pg total *ryr1a-ex6* TALEN RNAs (n = 24, red curves) were analyzed by HRMA at 1 dpf for the presence of newly induced mutations. Whereas no mutations at the *ryr3* locus were detected in these embryos, every injected embryo had mutations in the *ryr1a* and *ryr1b* genes.

As one test of the specificity of the TALENs used here, we generated TALENs to recognize a specific target sequence and measured the induction of mutations at very closely related sequences present in homologues of the targeted gene. The *ryr* genes encode related Ryanodine Receptor intracellular calcium release channels. We designed TALENs targeted to the *ryr3* or *ryr1a* genes and measured the induction of mutations in the homologous sequences present in the *ryr1a*, *ryr1b*, or *ryr3* genes. The *ryr3-ex5* TALEN recognizes a Left half-site of 19 nt and Right half-site of 18 nt (Figure S1). As illustrated in Figure 6A, the homologous sites in the *ryr1a* and *ryr1b* genes present a 3 base mismatch for the Left monomer and 4 base mismatch for the Right monomer of the *ryr3-ex5* TALEN. Although HRMA indicated every embryo injected with *ryr3-ex5* TALEN had induced mutations in *ryr3*, the embryos did not have detectable levels of mutations at the *ryr1a* or *ryr1b* loci (Figure 6B).

In a second experiment we analyzed the off-target activity of the *ryr1a-ex6* TALEN, which recognizes a Left half-site of 19 nt and Right half-site of 17 nt. As shown in Figure 6C, the homologous site in *ryr3* differs at 3 positions in both the Left and the Right half-sites, but the homologous site in *ryr1b* differs only at 2 positions in the Left half-site and presents a perfect match with the Right half-site. HRMA analysis revealed the *ryr1a-ex6* TALEN induced targeted mutations at the cognate locus in 100% of the injected embryos but failed to induce detectable mutations at the homologous *ryr3* target (Figure 6D). In contrast, the *ryr1a-ex6* TALEN did induce mutations in *ryr1b* gene of all of the injected embryos (Figure 6D). These results indicate the TALENs display high but not perfect sequence specificity under the conditions described here.

## Discussion

### Overview and implications

Sequence-specific TALENs that can target almost any zebrafish gene can be generated simply and rapidly using the target site design parameters, the reagents, and the cloning strategy described here. The TALENs generated using these reagents are very effective at inducing DSBs whose repair often produces mutations at the target sites. Using our standard methods to generate mutations at four different loci, we found virtually every embryo injected with TALEN mRNA harbored targeted mutations. As demonstrated with the *gol-ex2* TALEN, in some cases over half the genomes in a TALEN RNA-injected embryo may acquire mutations at targeted loci. Almost all treated animals acquire new germ line mutations, which are subsequently inherited in a stable Mendelian fashion. We have found many additional sites in the genome can be targeted with the ease and efficiency of the four genes described here: during the course of the current study we induced mutations at 19 of the 21 sites we attempted to target (90%). The genes that have been successfully targeted are present at many different locations in the genomes (Figure S1) including the *gol* locus, which is close to a telomere [33]. Further, as shown by the induction of *gol* mutations in early stage embryos (Figure S4), mutations can be induced in genes that are not being expressed. Given the modular mode of RVD repeat module binding, the flexibility in the design and the length of repeat arrays that can be generated, and the relatively few guidelines that restrict TALEN design, it appears TALENs can be used to induce loss-of-function mutations in almost every zebrafish gene.

The system we present here for constructing TALENs that are effective in the zebrafish is easy to implement: 1) it employs a very simple and rapid cloning strategy, using the Golden Gate cloning method system to assemble RVD repeats; 2) the RVD repeats are available in plasmids that can be used directly for Golden Gate cloning without need to PCR amplify or otherwise alter the repeat sequences; 3) the cloning strategy leads to accurate constructions of arrays (we typically analyze only 2 transformants from each cloning step); 4) the TALENs function as obligate heterodimers, increasing specificity of target sites recognized by the TALENs; and 5) all reagents are readily available through Addgene.

Although the studies presented here focus on induction of mutations in the zebrafish, our preliminary results indicate that TALENs assembled using the reagents described here are also effective at inducing mutations in *Drosophila* and mammalian cell culture systems.

We developed High Resolution Melt Analysis as a principal method for measuring the induction of mutations with TALENs in zebrafish embryos. HRMA can detect almost any newly arising polymorphism at a pre-specified region of the genome, and thus it allows detection of the large variety of sequence changes that may be produced by NHEJ repair. As HRMA can detect sequence alterations arising at any target site, this method of mutation detection does not bias or affect the choice of a target site and thus is an improvement over previous assays that detected loss of a restriction site. We show HRMA is an extremely sensitive method for detecting mutant genomes among a mixed population of genomes. Hence it is particularly useful for detecting TALEN-induced mutations, any one of which is likely to be present in only a subset of the cells of an embryo injected with TALEN RNAs. Furthermore, because HRMA is sensitive to the total fraction of mutant genomes in a mixture, it is particularly well-suited for detecting the heterogeneous set of mutations that is likely to arise in a single embryo. Finally, HRMA is simple to apply and does not involve manipulation of samples following PCR amplification. The entire procedure for generating amplicons and analyzing the thermostability of the heteroduplexes can be performed in less than 2 hours.

### Practical aspects of the use of TALENs to generate locus-specific mutations in zebrafish

We consider several practical issues concerning implementation of the methods presented here to induce mutations in the zebrafish: 1) the kinds of mutations generated and implications for target design considerations; 2) the frequency with which potential TALEN target sites can be identified; 3) the possibility that TALEN-injected embryos express mutant phenotypes; and 4) the ability of TALENs to induce unintended mutations.

Most of the mutations we have recovered from G0 germ lines are indels that affect ≤20 bp stretches of genomic sequence. Many of these sequence changes cause frameshift mutations. If TALENs are used with the goal of inducing null mutations in a targeted gene, it is best to target a region of the gene in which a frameshift mutation is likely to produce a protein product that lacks important functional elements. We routinely target the second or third exon of a gene. Among 40 genes for which we have designed TALENs, we have always been able to identify targets in the second and/or third exons of the genes using the design parameters presented in the Materials and Methods. Among searches of 116 stretches of (mostly coding) sequences with an average size 238 bp, we identified at least one suitable target in 77% of the sequences (the average size of the sequences without a best-fit target was 158 bp). Using the guidelines we suggest, optimal TALEN target sites can be identified for most genes.

The parameters that govern the specificity and activity of TALENs are not completely understood. It is clear from our studies and those of others that the Left and Right TALEN monomer components can bind at various distances from each other and still cooperate effectively to accomplish target site cleavage [27]. The spacer length for achieving optimal activity has not been determined, and we can only say the 14–17 bp spacer length we routinely use in the design of TALEN target sites consistently allows for effective target cleavage in the zebrafish. Importantly, the extreme minimum or maximum spacer distance at which some cleavage activity may occur has yet to be determined *in vivo*, an uncertainty that affects the identification of unintended sites in the genome that may be susceptible to TALEN activity.

As expected from previous work on the specificity and selectivity of TALE binding [25], [27], the TALENs function as highly sequence-specific nucleases. Given that we identify potential TALEN target sites on the basis of a reference genome, and as the common laboratory WT strains of zebrafish harbor polymorphisms, we have found it prudent to sequence genomic regions of the zebrafish used in any series of experiments to verify the existence of a presumed target site in the embryos that are injected with TALEN RNA. Furthermore, it should be noted HRMA can detect pre-existing polymorphisms, and thus we choose to inject embryos shown to be free of polymorphisms near the TALEN target site.

We routinely verify the *in vivo* activity of a TALEN before growing injected G0 embryos to adulthood. Typically we inject 1 cell stage embryos with different amounts (4 pg, 20 pg, 100 pg total RNA) of a 1∶1 mixture of Left TALEN and Right TALEN RNAs and measure the presence of mutations in individual 1 dpf embryos. In our experience to date, most animals that have evidence of mutations at 1 dpf will grow to become adults that transmit mutations through the germ line.

As demonstrated with the *gol-ex2* TALENs, cells with two mutant alleles can be induced following injection of 1 cell embryos with TALEN RNA. Mutations are induced in a mosaic fashion and using the conditions described here, it is rare that TALEN-injected embryos exhibit strong mutant phenotypes. It may be possible to augment the activity of the TALENs to uncover mutant phenotypes in G0 animals. In addition, it is worth considering the possibility that some mutations cannot survive in the germ line in a homozygous state. As a result, it may be desirable to raise G0 animals with sub-maximal levels of mutations. As demonstrated in our studies, the frequency of TALEN-induced mutations is a function of the amount of TALENs introduced into an animal. Thus, it is possible to raise animals that carry different mutation loads.

Finally, although we have not made extensive measurements of the frequency with which unintended sequences are recognized and cleaved by TALENs in these experiments, our studies indicate off-target mutations can occur but they are sufficiently infrequent so that they are unlikely to confound analysis of targeted gene function. We found TALENs failed to cleave potential target sites that differed at about 6 positions of a 36 nt binding target, but that targets differing at only 2 positions could be effectively cleaved. The effects of off-target mutations can be minimized by studying hetero-allelic combinations of targeted mutations derived from independently mutagenized G0 founders. In sum, the current conditions for TALEN-induced mutagenesis appear sufficient for uncovering the function of almost any selected gene in the zebrafish.

### Practical aspects of the use of HRMA to detect mutations

HRMA sensitively detects polymorphisms. To minimize detection of polymorphisms present in the backgrounds of WT fish, we typically amplify only a small region of the genome bordering the TALEN target site and analyze that for newly induced mutations. As a result of using small amplicons, we will be unable to detect some TALEN-induced mutations that delete primer-binding sites. As we only rarely observed deletions of >30 bp in the present studies, we believe the majority of TALEN-induced mutations can be identified with the methods described here. It is also possible to detect induced mutations by HRMA using larger amplicons. Finally, although the HRMA studies presented here were performed with a LightScanner (Idaho Technology), we have obtained identical results with similar sensitivity using an Eco Real-Time PCR System (Illumina). Additional instruments initially designed for qPCR analysis are capable of performing HRMA and have been used successfully to detect TALEN-induced mutations (Tatjana Piotrowski and Steven Leach, personal communication).

## Materials and Methods

### Ethics statement

All experiments were performed in accordance with, and under the supervision of, the Institutional Animal Care and Use Committee (IACUC) of the University of Utah, which is fully accredited by the AAALAC.

### Embryo culture and zebrafish stock maintenance

Wild type zebrafish *Danio rerio* were of the Tuebingen strain. Zebrafish were maintained under standard conditions and embryos were generated, cultured and staged as described [38], [39].

### TALEN target site design

Exon sequences identified from the Zv9 Zebrafish Genome Assembly were scanned for potential TALEN target sites, which were identified using the TALEN Targeter program at https://boglab.plp.iastate.edu/node/add/talen. The following parameters were used: 1) spacer length: 14–17; 2) repeat array length of 16–21; 3) apply all additional options that restrict target choice. Preference was given to target sites: 1) close to the 5′ terminus of the gene to maximize chances of inducing premature translation stop mutations and 2) not in the first exon in case alternative promoters exist. Target sequences that are unique in the genome should be chosen following BLAST analysis to determine that highly similar Left and Right binding sites in close proximity did not exist at other sites in the genome (see Figure 6).

### Creation of new backbone vectors

New final backbone vectors used to construct and express genes encoding Left and Right TALEN monomer components were generated here. The new backbone plasmids, pCS2TAL3DD and pCS2TAL3RR, were modified from pCS2-Flag-TTGZFP-FokI-DD and pCS2-HA-GAAZFP-FokI-RR plasmids [23]. First, to render the plasmids suitable for Golden Gate cloning, the single Esp3I restriction enzyme site (in the FokI nuclease domain) of each plasmid was changed from GAGACG to GCGCCG, a mutation that did not alter coding. Second, sequences encoding the ZFP domain were removed following KpnI and BamHI digestion, leaving a backbone vector with sequences 5′ to the KpnI site that provided a 5′UTR, start codon, NLS, and Flag or HA tags and sequences 3′ to the BamHI site that provided a heterodimeric FokI domain, translation termination codon, and SV40 polyA signal. Third, sequences derived from the *tal1c* gene and ready to accept an RVD repeat array by Golden Gate cloning were placed in frame at the KpnI and BamHI sites. The *tal1c* sequences were obtained from pTAL3 (sequence positions 1214–2210, www.addgene.org) using primers that added a KpnI site at the 5′ end (TAL3N153F-GTAGGATCCGGTACCGTGGATCTACGCACGCTCGG) and a BamHI site at the 3′ end (TAL3C63R-GTGGGATCCGGCAACGCGATGGGACG) of the amplified pTAL3 sequence. The amplified sequence encoded only a central portion of TAL1c, in which *lacZ* sequence had been substituted for the RVD repeat array. Cloning into the KpnI/BamHI sites of the backbone vector transferred sequence that provided 136 aa of TALE immediately N′-terminal to the RVD repeat array, an Esp3I restriction enzyme site, *lacZ* sequences, an Esp3I restriction enzyme site, and 63 aa of TALE immediately C′-terminal to the RVD repeat array. The TALE backbone truncations were designed after TALENs that previously had been shown to function well [27]. Golden Gate cloning of RVD repeat arrays into pCS2TAL3DD or pCS2TAL3RR results in replacement of the *lacZ* sequences with sequences encoding a designed RVD repeat array and yields a gene encoding an intact TALEN monomer. The pCS2TAL3-DD and pCS2TAL3-RR plasmids are available through Addgene (#37275 and #37276, respectively) with complete sequence information accessible at GenBank (accession numbers JX051360 and JX051361, respectively).

### TALEN assembly

The TALEN Golden Gate assembly system described in Cermark et al [30] was used with modifications. RVD repeat arrays were assembled exactly as described [30]. Plasmids providing RVD repeats for Golden Gate cloning are described in [30] and are available through Addgene. Briefly, two rounds of Golden Gate cloning assembly were used to generate a TALEN gene with *n* RVD repeat modules. First, two arrays were generated, corresponding to repeat modules 1–10 and 11 – *n-1*. Resulting vectors that acquired arrays were identified as white transformants on IPTG/X-gal plates. Correct assembly was determined first by the size of repeat array inserts liberated following XbaI and AflII restriction enzyme digestion and then sequencing of plasmids with the correct insert size. Second, the two arrays and sequences encoding the *n^th^* motif were transferred into the backbone vectors. RVD repeat array sequences were cloned into pCS2TAL3DD to generate a Left TALEN gene and into pCS2TAL3RR to generate a right TALEN gene. Backbone vectors that acquired arrays were identified as white transformants on IPTG/X-gal plates. Correct assembly was determined first by the size of the insert liberated by SphI and BamHI restriction enzyme digestion and second by sequencing junction regions.

### Injection of TALEN RNA into zebrafish embryos

5′-capped mRNA was generated by transcription *in vitro* of pCS2TAL3DD and pCS2TAL3RR TALEN plasmid templates that had been linearized with NotI (mMESSAGE mMACHINE SP6 kit, Ambion/Invitrogen). Equal amounts of Left and Right TALEN mRNA were injected together into the cytoplasm of 1 cell stage zebrafish embryos.

### Genomic DNA extraction

To prepare genomic DNA from embryos, individual 1 or 2 dpf embryos were incubated in 50 ul DNA extraction buffer [10 mM Tris-HCl (pH 8.0), 1 mM EDTA, 50 mM KCl, 0.3% Tween-20, 0.3% NP-40] containing 500 ug/ml proteinase K at 55°C, 2 h. The reaction was terminated by incubation at 99°C, 5 min. The average gDNA concentration was roughly 60 ng/ul.

### High Resolution Melt Analysis (HRMA)

To detect TALEN-induced mutations by HRMA, a 90–120 bp amplicon that included the entire genomic target site was generated. Primers flanking the target site were used to amplify the genomic region in a 10 ul PCR reaction containing: 1 ul embryonic gDNA, 1X LightScanner Master Mix (containing the LC Green Plus dye, Idaho Technology), 200 uM each dNTP, and 200 nM each Forward and Reverse primers (see Table S1). Amplification/duplex formation conditions were: denaturation at 95°C, 3 min; 50 cycles {95°C, 30 s–70°C, 18 s}; denaturation at 95°C, 30 s; renaturation at 25°C, 30 s; 10°C. HRMA data was collected on a LightScanner (Idaho Technology) and analyzed using the LightScanner Call-IT Software.

## Supporting Information

Figure S1 TALENs and TALEN target sites used. (A) Sequences of the TALEN target sites are indicated, with the Left and Right TALEN monomer binding sites highlighted in color. In our studies, each monomer binding site begins with Thymine (blue), which is presumably contacted by the N-terminal domain of the TALEN encoded by the backbone vector and which appears to contribute to overall binding. The sequences recognized by arrays of RVD repeat modules are indicated in red. The RVD modules used to recognize each nucleotide are indicated. The name of each TALEN designates the gene and the exon (according to Ensemble zebrafish Zv9 assembly) that is targeted. The relative location of each target gene is depicted on its chromosome, with the position of the centromere indicated as a dot. (B) For each target site used in this study, the nucleotide lengths of target site domains are tabulated, including the length of each Left and Right RVD repeat array binding site as well as the spacer region between binding sites.
(TIF)

Figure S2 pCS2TAL3DD and pCS2TAL3RR. Schematic representation of the pCS2TAL3DD and pCS2TAL3RR vectors generated in this study to build and express genes encoding the Left and Right TALEN monomers. The plasmid backbone (solid black line), the simian IE94 cytomegalovirus eukaryotic enhancer/promoter (CMV), the recognition sequence used by the prokaryotic SP6 RNA polymerase (SP6), and the polyadenylation signal sequence derived from SV40 (SV40pA) were derived from the CS2+ plasmids (http://sitemaker.umich.edu/dlturner.vectors). Other domains indicated encode: a nuclear localization signal (NLS); the FLAG epitope (Flag); the hemagglutinin epitope (HA); truncated N-terminus and C-terminus (TAL-N′ and TAL-C′) sequences derived from pTAL3; nuclease domains of the FokI restriction enzyme with DD and RR mutations (FokI (DD) and FokI (RR)). Significant restriction enzyme sites are indicated: KpnI (Kp), Esp3I (Esp), BamHI (Bm), XbaI (Xb), and NotI (Nt). The pCS2TAL3-DD and pCS2TAL3-RR plasmids are available through Addgene (#37275 and #37276, respectively) with complete sequence information accessible at GenBank (accession numbers JX051360 and JX051361, respectively).
(TIF)

Figure S3 HRMA can detect the presence of a 4 bp insertion mutation among WT genomes. (A, B) HRMA analysis to detect *lef1* genomic sequences. PCR amplicons were generated from template genomes that were either WT *(lef1^+/+^)*, heterozygous for a 4 bp insertion *(lef1^+4/+^)*, or mixtures of WT and heterozygous genomes. Fractions indicate the relative abundance of mutant haploid genomes in the mixtures. A constant total amount of genomic template DNA and primers was used to generate each set of amplicons. One set of experimental data is plotted in two different ways: (A) Each amplicon melt curve is plotted as a function of fraction of Normalized Fluorescence (normalized so that the maximal fluorescence for each amplicon is defined as 1.0) vs. Temperature; (B) For each amplicon the difference (Δ Fluorescence) between the fraction of maximal fluorescence of each amplicon vs. that of a WT amplicon is plotted as a function of Temperature. Δ Fluorescence plots are used commonly to highlight deviations from WT melt profiles. LightScanner Call-IT Software (Idaho Technology) was used to identify melt curves that differed significantly from WT. HRMA detected the lef1 4 bp insertion allele at significantly detectable levels when it was present as only 1/70^th^ of the total haploid genomes.
(TIF)

Figure S4 Time course of induction of mutations with TALENs. WT embryos were either not injected or injected at the 1 cell stage with gol-ex2 TALEN RNAs and analyzed by HRMA at 3, 6, or 24 hpf for the presence of targeted mutations. LightScanner Call-IT Software (Idaho Technology) was used to identify melt curves that differed significantly from WT. 3 hpf embryos contain about 1000 cells, and thus yield limited gDNA, only a portion of which is used as template for each HRMA analysis. The HRMA melt profiles of the amplicons derived from the limited gDNA template have increased variability as compared with standard conditions of analysis. Whereas every injected embryo had newly induced mutations that could be detected at 6 or 24 hpf, the melt profiles of some 3 hpf injected embryos (blue) could not be distinguished unambiguously from the melt profiles of control WT embryos (grey). Furthermore, in contrast to the melt profiles of the injected 6 or 24 hpf embryos, the melt profiles of the 3 hpf embryos with mutations (red) diverged only modestly from that of the WT curves, reflecting a relatively lower abundance of mutant genomes present in the 3 hpf embryos.
(TIF)

Table S1 Primers used for HRMA. Forward (F) and Reverse (R) primers used to amplify the genomic regions surrounding targeted sequences of the *golden*, *ryr3*, *ryr1a*, *ryr1b*, and *lef1* loci.
(DOCX)

Table S2 Induction of *gol*-like mutants by *gol-ex2* TALEN. One cell stage embryos were injected with *gol-ex2* TALEN RNA and embryos were analyzed at 2 dpf. Embryos with ≤20 total darkly pigmented cells, including melanophores and RPE cells, were scored as ‘*gol*-like embryos’.
(DOCX)

Table S3 TALENs induce mutations without perturbing development. One cell stage embryos were injected with a total of 100 pg mRNA encoding Left and Right TALEN monomer components directed at the indicated target loci. Uninjected control and injected embryos were inspected for normal morphology and viable appearance at 1 dpf.
(DOCX)

Table S4 Distribution of mutations in germ lines of *gol-ex2* TALEN-injected founders. One cell stage embryos were injected with *gol-ex2* TALEN RNA and raised to adulthood. G0 adult founders were mated with WT partners. To identify newly induced mutations in the germ lines of the G0 founders and to estimate the fractional representation of each mutation within a germ line, individual 1–2 dpf F1 embryos were analyzed for presence of *gol* mutations by HRMA. *n* is the number of F1 embryos analyzed.
(DOCX)

Table S5 Distribution of mutations in germ lines of *ryr3-ex5* TALEN-injected founders. One cell stage embryos were injected with *ryr3-ex5* TALEN RNA and raised to adulthood. G0 adult founders were mated with WT partners. To identify newly induced mutations in the germ lines of the G0 founders and to estimate the fractional representation of each mutation within a germ line, individual 1–2 dpf F1 embryos were analyzed for presence of *ryr3* mutations by HRMA. *n* is the number of F1 embryos analyzed.
(DOCX)

Table S6 Distribution of mutations in germ lines of *tbx6-ex3* TALEN-injected founders. One cell stage embryos were injected with *tbx6-ex3* TALEN RNA and raised to adulthood. G0 adult founders were mated with WT partners. To identify newly induced mutations in the germ lines of the G0 founders and to estimate the fractional representation of each mutation within a germ line, individual 1–2 dpf F1 embryos were analyzed for presence of *tbx6* mutations by HRMA. *n* is the number of F1 embryos analyzed.
(DOCX)

Table S7 Distribution of mutations in germ lines of *ryr1a-ex6* TALEN-injected founders. One cell stage embryos were injected with *ryr1a-ex6* TALEN RNA and raised to adulthood. G0 adult founders were mated with WT partners. To identify newly induced mutations in the germ lines of the G0 founders and to estimate the fractional representation of each mutation within a germ line, individual 1–2 dpf F1 embryos were analyzed for presence of *ryr1a* mutations by HRMA. *n* is the number of F1 embryos analyzed.
(DOCX)

Table S8 Distribution of mutations among F1 adults descended from TALEN-injected founders. Each F1 family was produced from a mating between a G0 founder and WT partners. The name of each F1 family indicates the G0 founder, listed in Tables S5, S6, S7. Fin biopsies were performed on 2–3 month heterozygous F1 adults for genotyping. Mutant alleles were detected by HRMA of gDNA isolated from the fin biopsies. In many cases the mutant alleles were sequenced and the sequence change is indicated. n.d., no data.
(DOCX)
