# Supplementary figures and images for: Simple Methods for Generating and Detecting Locus-Specific Mutations Induced with TALENs in the Zebrafish Genome
Source: PLoS Genet. 2012 Aug 16;8(8):e1002861. doi: 10.1371/journal.pgen.1002861 (PMC3420959; doi:10.1371/journal.pgen.1002861)

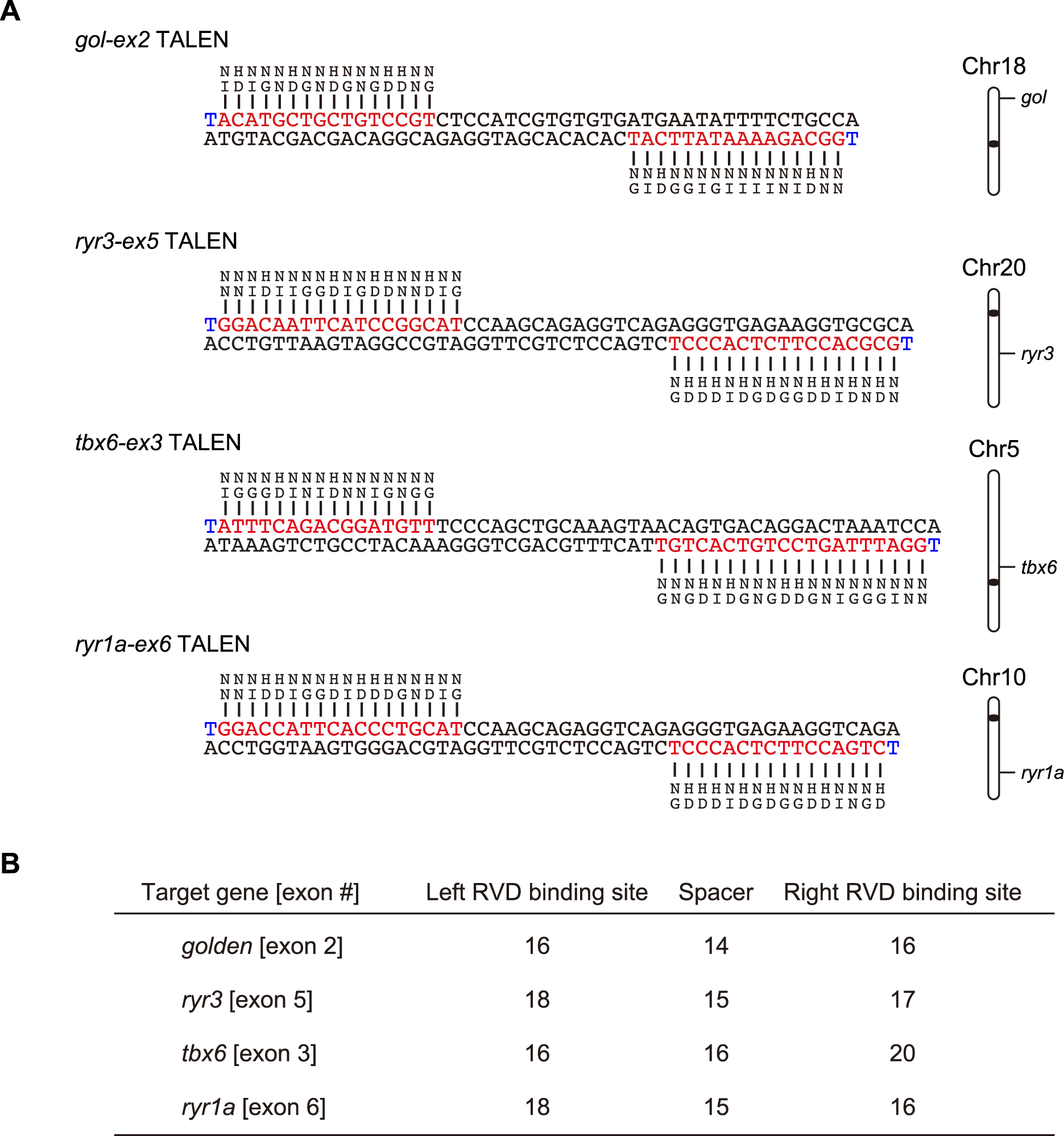

Supplement: Figure S1 — TALENs and TALEN target sites used. (A) Sequences of the TALEN target sites are indicated, with the Left and Right TALEN monomer binding sites highlighted in color. In our studies, each monomer binding site begins with Thymine (blue), which is presumably contacted by the N-terminal domain of the TALEN encoded by the backbone vector and which appears to contribute to overall binding. The sequences recognized by arrays of RVD repeat modules are indicated in red. The RVD modules used to recognize each nucleotide are indicated. The name of each TALEN designates the gene and the exon (according to Ensemble zebrafish Zv9 assembly) that is targeted. The relative location of each target gene is depicted on its chromosome, with the position of the centromere indicated as a dot. (B) For each target site used in this study, the nucleotide lengths of target site domains are tabulated, including the length of each Left and Right RVD repeat array binding site as well as the spacer region between binding sites. (TIF) [file pgen.1002861.s001.tif]

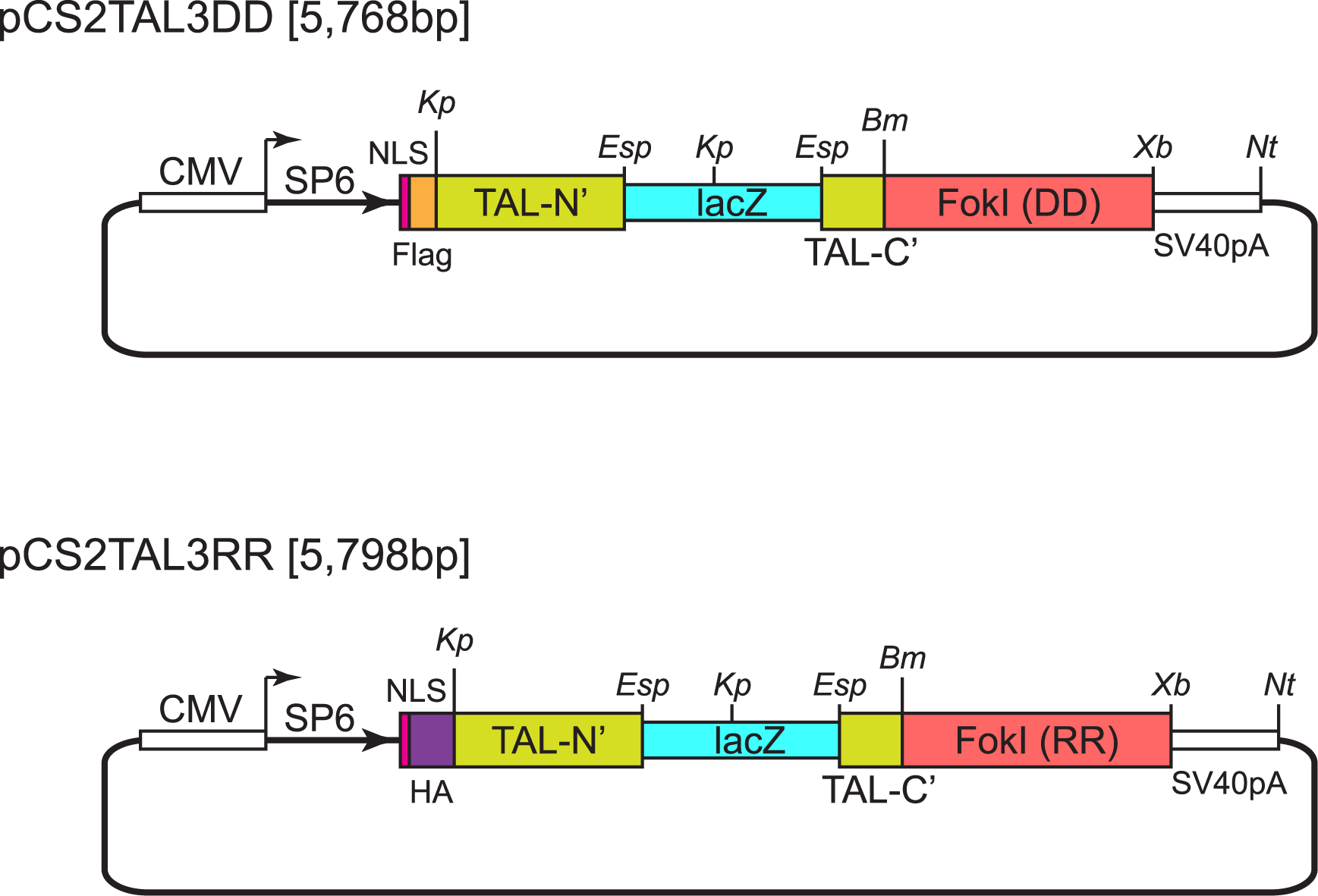

Supplement: Figure S2 — pCS2TAL3DD and pCS2TAL3RR. Schematic representation of the pCS2TAL3DD and pCS2TAL3RR vectors generated in this study to build and express genes encoding the Left and Right TALEN monomers. The plasmid backbone (solid black line), the simian IE94 cytomegalovirus eukaryotic enhancer/promoter (CMV), the recognition sequence used by the prokaryotic SP6 RNA polymerase (SP6), and the polyadenylation signal sequence derived from SV40 (SV40pA) were derived from the CS2+ plasmids (http://sitemaker.umich.edu/dlturner.vectors). Other domains indicated encode: a nuclear localization signal (NLS); the FLAG epitope (Flag); the hemagglutinin epitope (HA); truncated N-terminus and C-terminus (TAL-N′ and TAL-C′) sequences derived from pTAL3; nuclease domains of the FokI restriction enzyme with DD and RR mutations (FokI (DD) and FokI (RR)). Significant restriction enzyme sites are indicated: KpnI (Kp), Esp3I (Esp), BamHI (Bm), XbaI (Xb), and NotI (Nt). The pCS2TAL3-DD and pCS2TAL3-RR plasmids are available through Addgene (#37275 and #37276, respectively) with complete sequence information accessible at GenBank (accession numbers JX051360 and JX051361, respectively). (TIF) [file pgen.1002861.s002.tif]

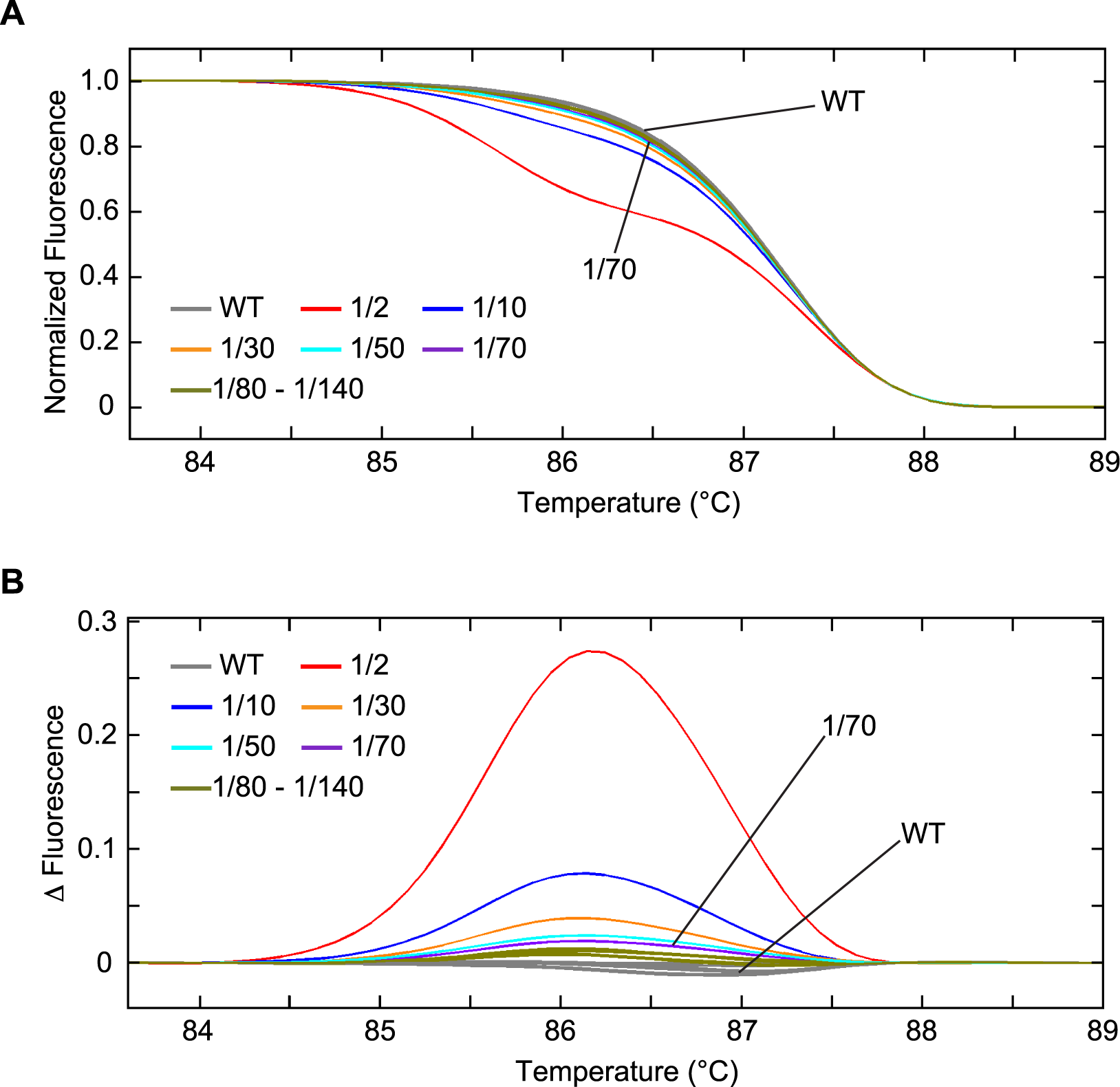

Supplement: Figure S3 — HRMA can detect the presence of a 4 bp insertion mutation among WT genomes. (A, B) HRMA analysis to detect lef1 genomic sequences. PCR amplicons were generated from template genomes that were either WT (lef1+/+), heterozygous for a 4 bp insertion (lef1+4/+), or mixtures of WT and heterozygous genomes. Fractions indicate the relative abundance of mutant haploid genomes in the mixtures. A constant total amount of genomic template DNA and primers was used to generate each set of amplicons. One set of experimental data is plotted in two different ways: (A) Each amplicon melt curve is plotted as a function of fraction of Normalized Fluorescence (normalized so that the maximal fluorescence for each amplicon is defined as 1.0) vs. Temperature; (B) For each amplicon the difference (Δ Fluorescence) between the fraction of maximal fluorescence of each amplicon vs. that of a WT amplicon is plotted as a function of Temperature. Δ Fluorescence plots are used commonly to highlight deviations from WT melt profiles. LightScanner Call-IT Software (Idaho Technology) was used to identify melt curves that differed significantly from WT. HRMA detected the lef1 4 bp insertion allele at significantly detectable levels when it was present as only 1/70th of the total haploid genomes. (TIF) [file pgen.1002861.s003.tif]

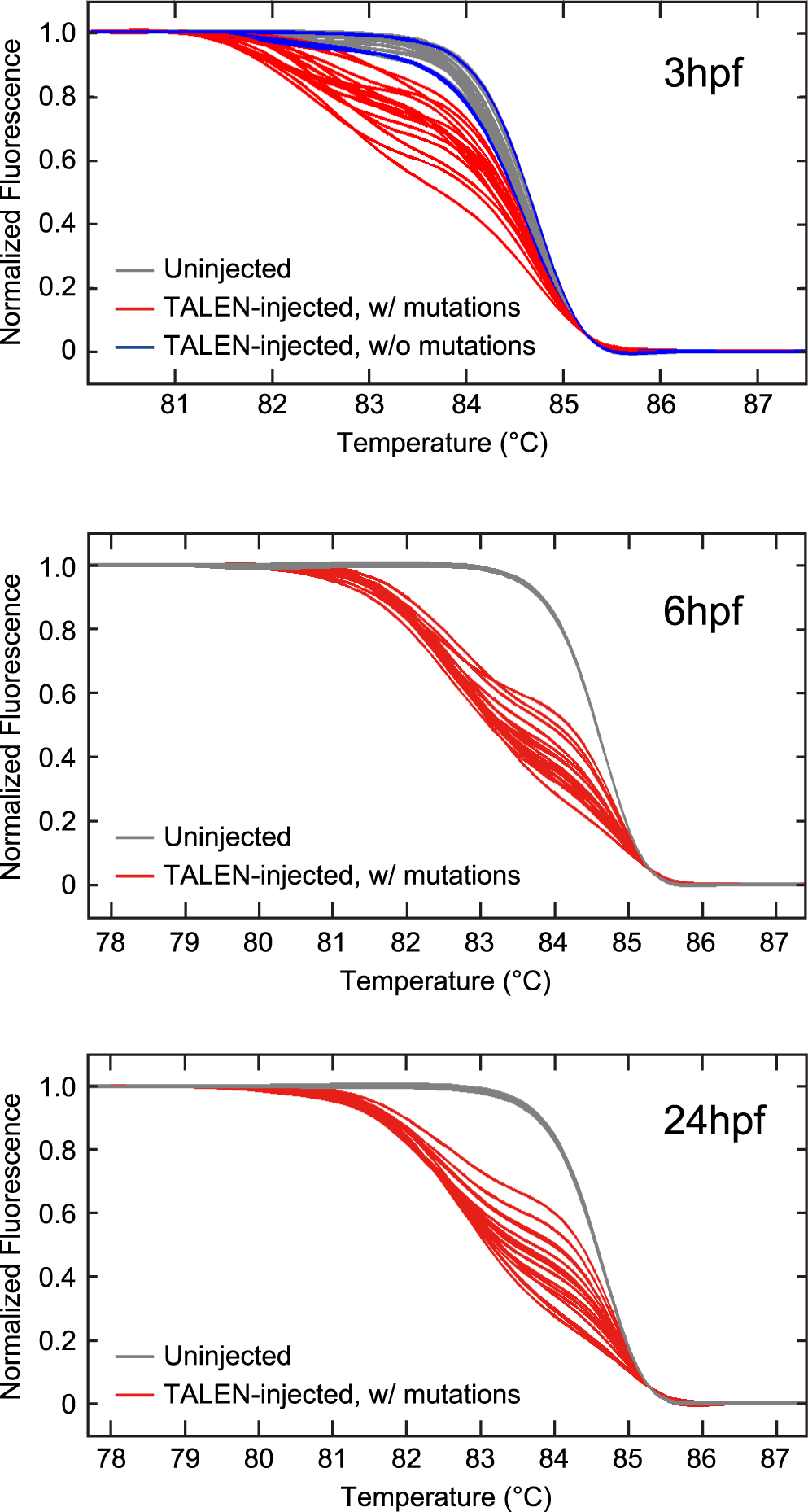

Supplement: Figure S4 — Time course of induction of mutations with TALENs. WT embryos were either not injected or injected at the 1 cell stage with gol-ex2 TALEN RNAs and analyzed by HRMA at 3, 6, or 24 hpf for the presence of targeted mutations. LightScanner Call-IT Software (Idaho Technology) was used to identify melt curves that differed significantly from WT. 3 hpf embryos contain about 1000 cells, and thus yield limited gDNA, only a portion of which is used as template for each HRMA analysis. The HRMA melt profiles of the amplicons derived from the limited gDNA template have increased variability as compared with standard conditions of analysis. Whereas every injected embryo had newly induced mutations that could be detected at 6 or 24 hpf, the melt profiles of some 3 hpf injected embryos (blue) could not be distinguished unambiguously from the melt profiles of control WT embryos (grey). Furthermore, in contrast to the melt profiles of the injected 6 or 24 hpf embryos, the melt profiles of the 3 hpf embryos with mutations (red) diverged only modestly from that of the WT curves, reflecting a relatively lower abundance of mutant genomes present in the 3 hpf embryos. (TIF) [file pgen.1002861.s004.tif]
