# Supplementary material for: Simple Methods for Generating and Detecting Locus-Specific Mutations Induced with TALENs in the Zebrafish Genome
Source: PLoS Genet. 2012 Aug 16;8(8):e1002861. doi: 10.1371/journal.pgen.1002861 (PMC3420959; doi:10.1371/journal.pgen.1002861)
Supplement: Table S1 — Primers used for HRMA. Forward (F) and Reverse (R) primers used to amplify the genomic regions surrounding targeted sequences of the golden, ryr3, ryr1a, ryr1b, and lef1 loci. (DOCX) [file pgen.1002861.s005.docx]

**Table S1. Primers used for HRMA**

| **Primer name** | **Sequence (5’ to 3’)** |
| --- | --- |
| golden-F | CAGGAGAGGAAAGATGGAGGAA |
| golden-R | GCTGATGACCTCCAGAGATGG |
| ryr3-F | GGGAAGCTTGTTGGTGGACA |
| ryr3-R | GGACACGCTGACCAAGATGA |
| tbx6-F | CGGTGTGCTTTTATTTCAGACG |
| tbx6-R | GCACCATGTCCATTATCACCAC |
| ryr1a-F | CTTCTAGGTGAGGCCTGTTGGT |
| ryr1a-R | GGACACGCTGACCAGAATAATG |
| ryr1b-F | ACTGTGTTCAGGGGAAGCATGT |
| ryr1b-R | GCTGACAAGGATCAGGTCGTCT |
| lef1-F | GCACAAGGAGCAAATCTTCG |
| lef1-R | TGATTTCTGTCTCGTTGACCA |

Forward (F) and Reverse (R) primers used to amplify the genomic regions surrounding targeted sequences of the *golden*, *ryr3*, *ryr1a*, *ryr1b*, and *lef1* loci.
