# Supplementary material for: Simple Methods for Generating and Detecting Locus-Specific Mutations Induced with TALENs in the Zebrafish Genome
Source: PLoS Genet. 2012 Aug 16;8(8):e1002861. doi: 10.1371/journal.pgen.1002861 (PMC3420959; doi:10.1371/journal.pgen.1002861)
Supplement: Table S2 — Induction of gol-like mutants by gol-ex2 TALEN. One cell stage embryos were injected with gol-ex2 TALEN RNA and embryos were analyzed at 2 dpf. Embryos with ≤20 total darkly pigmented cells, including melanophores and RPE cells, were scored as ‘gol-like embryos’. (DOCX) [file pgen.1002861.s006.docx]

**Table S2. Induction of *gol*-like mutants by *gol-ex2* TALEN**

| **Amount TALEN RNA injected** | **# embryos analyzed** | **# *gol*-like embryos (%)** |
| --- | --- | --- |
| 100pg | 386 | 1 (0.26%) |
| 200pg | 169 | 3 (1.8%) |
| 400pg | 107 | 5 (4.7%) |

One cell stage embryos were injected with *gol-ex2* TALEN RNA and embryos were analyzed at 2 dpf. Embryos with < 20 total darkly pigmented cells, including melanophores and RPE cells, were scored as ‘*gol*-like embryos’.
