# Supplementary material for: Simple Methods for Generating and Detecting Locus-Specific Mutations Induced with TALENs in the Zebrafish Genome
Source: PLoS Genet. 2012 Aug 16;8(8):e1002861. doi: 10.1371/journal.pgen.1002861 (PMC3420959; doi:10.1371/journal.pgen.1002861)
Supplement: Table S3 — TALENs induce mutations without perturbing development. One cell stage embryos were injected with a total of 100 pg mRNA encoding Left and Right TALEN monomer components directed at the indicated target loci. Uninjected control and injected embryos were inspected for normal morphology and viable appearance at 1 dpf. (DOCX) [file pgen.1002861.s007.docx]

**Table S3. TALENs induce mutations without perturbing development**

| **Targeted locus** | **Fraction (%) normal embryos** |
| --- | --- |
| Uninjected controls | 617/618 (99.8%) |
| *golden* [exon 2] | 299/309 (96.8%) |
| *ryr3* [exon 5] | 175/190 (92.1%) |
| *tbx6* [exon 6] | 189/192 (98.4%) |

One cell stage embryos were injected with a total of 100 pg mRNA encoding Left and Right TALEN monomer components directed at the indicated target loci. Uninjected control and injected embryos were inspected for normal morphology and viable appearance at 1 dpf.
