# Supplementary material for: Simple Methods for Generating and Detecting Locus-Specific Mutations Induced with TALENs in the Zebrafish Genome
Source: PLoS Genet. 2012 Aug 16;8(8):e1002861. doi: 10.1371/journal.pgen.1002861 (PMC3420959; doi:10.1371/journal.pgen.1002861)
Supplement: Table S4 — Distribution of mutations in germ lines of gol-ex2 TALEN-injected founders. One cell stage embryos were injected with gol-ex2 TALEN RNA and raised to adulthood. G0 adult founders were mated with WT partners. To identify newly induced mutations in the germ lines of the G0 founders and to estimate the fractional representation of each mutation within a germ line, individual 1–2 dpf F1 embryos were analyzed for presence of gol mutations by HRMA. n is the number of F1 embryos analyzed. (DOCX) [file pgen.1002861.s008.docx]

**Table S4. Distribution of mutations in germ lines of *gol-ex2* TALEN-injected founders**

| **G0 founder** | **Allele** | **Frequency among**  ***n* gametes** | |
| --- | --- | --- | --- |
| f1 | WT (+/+) | 100% | (*n* = 24) |
| f2 | *gol*-#1 | 8.3% | (n = 12) |
|  | *gol*-#2 | 8.3% |  |
|  | WT (+/+) | 83.3% |  |
| f3 | *gol*-#3 | 29.2% | (n = 24) |
|  | *gol*-#4 | 8.3% |  |
|  | WT (+/+) | 62.5% |  |
| f4 | *gol*-#5 | 25.0% | (*n* = 24) |
|  | *gol*-#6 | 29.2% |  |
|  | WT (+/+) | 45.8% |  |
| f5 | *gol*-#7 | 12.5% | (*n* = 24) |
|  | *gol*-#8 | 8.3% |  |
|  | *gol*-#9 | 4.2% |  |
|  | WT (+/+) | 75.0% |  |
| f6 | *gol*-#10 | 25.0% | (*n* = 24) |
|  | *gol*-#11 | 8.3% |  |
|  | *gol*-#12 | 8.3% |  |
|  | WT (+/+) | 58.3% |  |
| f7 | *gol*-#13 | 25.0% | (*n* = 24) |
|  | *gol*-#14 | 12.5% |  |
|  | *gol*-#15 | 4.2% |  |
|  | WT (+/+) | 58.3% |  |
| f8 | *gol*-#16 | 8.3% | (*n* = 24) |
|  | WT (+/+) | 91.7% |  |
| m1 | *gol*-#17 | 20.8% | (*n* = 24) |
|  | *gol*-#18 | 4.2% |  |
|  | WT (+/+) | 75% |  |
| m2 | *gol*-#19 | 20.8% | (*n* = 24) |
|  | *gol*-#20 | 20.8% |  |
|  | WT (+/+) | 58.3% |  |
| m3 | *gol*-#21 | 25.0% | (*n* = 24) |
|  | *gol*-#22 | 4.2% |  |
|  | WT (+/+) | 70.8% |  |
| m4 | *gol*-#23 | 29.2% | (*n* = 24) |
|  | *gol*-#24 | 12.5% |  |
|  | *gol*-#25 | 4.2% |  |
|  | WT (+/+) | 54.2% |  |
| m5 | *gol*-#26 | 45.8% | (*n* = 24) |
|  | *gol*-#27 | 50.0% |  |
|  | *gol*-#28 | 4.2% |  |
|  | WT (+/+) | 0 |  |
| m6 | *gol*-#29 | 4.2% | (*n* = 24) |
|  | WT (+/+) | 95.8% |  |
| m7 | *gol*-#30 | 41.7% | (*n* = 24) |
|  | *gol*-#31 | 37.5% |  |
|  | *gol*-#32 | 4.2% |  |
|  | WT (+/+) | 16.7% |  |

One cell stage embryos were injected with *gol-ex2* TALEN RNA and raised to adulthood. G0 adult founders were mated with WT partners. To identify newly induced mutations in the germ lines of the G0 founders and to estimate the fractional representation of each mutation within a germ line, individual 1- 2 dpf F1 embryos were analyzed for presence of *gol* mutations by HRMA. *n* is the number of F1 embryos analyzed.
