# Supplementary material for: Simple Methods for Generating and Detecting Locus-Specific Mutations Induced with TALENs in the Zebrafish Genome
Source: PLoS Genet. 2012 Aug 16;8(8):e1002861. doi: 10.1371/journal.pgen.1002861 (PMC3420959; doi:10.1371/journal.pgen.1002861)
Supplement: Table S8 — Distribution of mutations among F1 adults descended from TALEN-injected founders. Each F1 family was produced from a mating between a G0 founder and WT partners. The name of each F1 family indicates the G0 founder, listed in Tables S5, S6, S7. Fin biopsies were performed on 2–3 month heterozygous F1 adults for genotyping. Mutant alleles were detected by HRMA of gDNA isolated from the fin biopsies. In many cases the mutant alleles were sequenced and the sequence change is indicated. n.d., no data. (DOCX) [file pgen.1002861.s012.docx]

**Table S8. Distribution of mutations among F1 adults descended from**

**TALEN-injected founders**

| **Targeted gene** | **F1 family** | **Allele** | **Frequency among *n* F1 adult offspring** | | **Mutation** |
| --- | --- | --- | --- | --- | --- |
| *ryr3* | m4 | *ryr3^m4-3^* | 19.0% | (*n* = 42) | ∆10bp |
|  |  | *ryr3^m4-4^* | 7.1% |  | ∆6bp |
|  |  | *ryr3^m4-37^* | 2.4% |  | n.d. |
|  |  | WT | 71.4% |  | +/+ |
|  | f9 | *ryr3^f9-11^* | 25.0% | (*n* = 56) | ∆14bp |
|  |  | WT | 75.0% |  | +/+ |
| *tbx6* | m4 | *tbx6^m4-13^* | 27.0% | (*n* = 37) | ∆4bp, +1bp |
|  |  | *tbx6^m4-16^* | 18.9% |  | ∆8bp |
|  |  | *tbx6^m4-10^* | 16.2% |  | ∆4bp |
|  |  | *tbx6^m4-11^* | 2.7% |  | ∆16bp |
|  |  | *tbx6^m4-3^* | 2.7% |  | +3bp |
|  |  | WT | 32.4% |  | +/+ |
|  | f5 | *tbx6^f5-1^* | 25.0% | (*n* = 16) | ∆8bp, +5bp |
|  |  | WT | 75.0% |  | +/+ |
| *ryr1a* | m3 | *ryr1a^m3-8^* | 42.4% | (*n* = 59) | ∆4bp |
|  |  | *ryr1a^m3-51^* | 6.8% |  | n.d. |
|  |  | *ryr1a^m3-37^* | 1.7% |  | n.d. |
|  |  | WT | 49.2% |  | +/+ |
|  | m9 | *ryr1a^m9-1^* | 50.0% | (*n* = 36) | ∆11bp |
|  |  | *ryr1a^m9-17^* | 5.6% |  | ∆6bp, +3bp |
|  |  | *ryr1a^m9-14^* | 2.8% |  | n.d. |
|  |  | WT | 41.7% |  | +/+ |

Each F1 family was produced from a mating between a G0 founder and WT partners. The name of each F1 family indicates the G0 founder, listed in Tables S5 – S7. Fin biopsies were performed on 2 – 3 month heterozygous F1 adults for genotyping. Mutant alleles were detected by HRMA of gDNA isolated from the fin biopsies. In many cases the mutant alleles were sequenced and the sequence change is indicated. n.d., no data.
